# The NAM/ATAF1/2/CUC2 transcription factor PpNAC.A59 enhances *PpERF.A16* expression to promote ethylene biosynthesis during peach fruit ripening

**DOI:** 10.1038/s41438-021-00644-6

**Published:** 2021-10-01

**Authors:** Zhi-Hua Guo, You-Jia Zhang, Jia-Long Yao, Zhi-Hua Xie, Yu-Yan Zhang, Shao-Ling Zhang, Chao Gu

**Affiliations:** 1 College of Horticulture/State Key Laboratory of Crop Genetics and Germplasm Enhancement, Nanjing Agricultural University, 210095 Nanjing, China; 2 New Zealand Institute of Plant & Food Research Ltd, Private Bag 92169, Auckland, 1142 New Zealand; 3 Institute of Pomology, Jiangsu Academy of Agricultural Sciences/Jiangsu Key Laboratory for Horticultural Crop Genetic Improvement, 210014 Nanjing, China

**Keywords:** Plant hormones, Transcription

## Abstract

Peach is a typical climacteric fruit that releases ethylene during fruit ripening. Several studies have been conducted on the transcriptional regulation of ethylene biosynthesis in peach fruit. Herein, an ethylene response factor, PpERF.A16, which was induced by exogenous ethylene, could enhance ethylene biosynthesis by directly inducing the expression of *1-aminocyclopropane-1-carboxylic acid synthase* (*PpACS1*) and *1-aminocyclopropane-1-carboxylic acid oxidase* (*PpACO1*) genes. Moreover, the NAM/ATAF1/2/CUC2 (NAC) transcription factor (TF) *PpNAC.A59* was coexpressed with *PpERF.A16* in all tested peach cultivars. Interestingly, PpNAC.A59 can directly interact with the promoter of *PpERF.A16* to induce its expression but not enhance *LUC* activity driven by any promoter of *PpACS1* or *PpACO1*. Thus, PpNAC.A59 can indirectly mediate ethylene biosynthesis via the NAC-ERF signaling cascade to induce the expression of both *PpACS1* and *PpACO1*. These results enrich the genetic network of fruit ripening in peach and provide new insight into the ripening mechanism of other perennial fruits.

## Introduction

Fruit ripening is the last stage of the fruit developmental process and is accompanied by a series of physiological and metabolic changes in color, texture, flavor, and nutritional compounds^1^. Fleshy fruits are classically defined as climacteric or nonclimacteric; the former exhibit a burst of ethylene production and respiration during ripening, while the latter do not^2^. Ethylene is a major signaling molecule that controls fruit ripening in climacteric fruits^3^ and plays an important role in the fruit ripening of nonclimacteric fruits^4,5^.

Ethylene biosynthesis and signal transduction have been widely studied in angiosperms^6–8^. Ethylene biosynthesis is catalyzed by two enzymes, 1-aminocyclopropane-1-carboxylic acid synthase (ACS), and oxidase (ACO). Synthesized ethylene activates the expression of ripening-related genes through transcription factors (TFs), ETHYLENE INSENSITIVE-like factors and ethylene responsive factors (ERFs). In tomato, LeERF1 promotes fruit ripening^9^, while SlERF6 delays fruit ripening^10^. In perennial fruit trees, MaERF9, MdERF3, and PbERF24 induce ethylene biosynthesis during fruit ripening in banana, apple, and pear, respectively^11–13^. In contrast, MaERF11, MdERF2, and CpERF9 inhibit ethylene biosynthesis during fruit ripening in banana, apple, and papaya, respectively^11,12,14^. Notably, apple MdERF3 is the downstream target of MdERF2, MdMYC2, and MdARF5^12,15,16^. These findings suggest that ERFs are important in ethylene biosynthesis during fruit ripening.

In addition to ERF, other TFs, such as NAM/ATAF1/2/CUC2 (NAC), MADS-box, homeobox, apetala 2, SQUAMOSA promoter binding protein (SBP), auxin response factor (ARF), and zinc-finger proteins, are also involved in mediating ethylene biosynthesis in climacteric fruits^8,17–20^. Among the NAC TFs, NOR, SlNAC4, and NAC-like 1 are positive regulators of fruit ripening^21–23^, while SlNAC1 is a transcriptional repressor of ripening-related genes in tomato^24^. AdNAC6 and AdNAC7 can bind to the *AdACS1* and *AdACO1* promoters to stimulate its expression and increase ethylene production in kiwifruit^25^. MaNAC1 and MaNAC2 can directly interact with ethylene signaling components, such as ETHYLENE INSENSITIVE, in banana^26^. CmNAC-NOR is located in a quantitative trait locus for climacteric ripening and is likely associated with ethylene biosynthesis and fruit ripening in melon^27^. Moreover, a heterodimer of two NACs can activate the transcription of an MYB TF^28^, indicating that the NAC TF is probably an upstream factor that regulates the expression of downstream TF(s). These findings suggest that NAC could be involved in regulating ethylene biosynthesis by directly and/or indirectly inducing ethylene biosynthesis genes. This suggestion is supported by previous reports, in which three MADS-box TFs, RIN, FUL1, and FUL2, can mediate the expression of *ACS* genes and their upstream TFs, including *RIN*, *NOR*, and *CNR* (an SBP TF)^29,30^.

Peach (*Prunus persica*) emits a large amount of ethylene during fruit ripening^31,32^. Exogenous ethylene accelerates fruit softening and induces the expression of softening-related genes^33^, such as *endopolygalacturonase* (*PpendoPGM*) controlling the melting flesh phenotype in the clingstone melting flesh (CMF) peach fruit^34^. Moreover, ethylene biosynthesis and signal transduction in peach can be interrupted by aminoethoxyvinylglycine and 1-methylcyclopropene (1-MCP), respectively, leading to the delay of fruit ripening^35,36^. Therefore, ethylene is crucial for catalyzing fruit ripening in peach. The genes *PpACS1* and *PpACO1*, which encode the two catalytic enzymes for ethylene biosynthesis, have been identified in peach fruit^37–39^. Two TFs, PpHB.G7 and PpTCP.A2, were identified to be involved in peach fruit ripening^40,41^. PpHB.G7 can bind to the *PpACS1* and *PpACO1* promoters to induce the expression of both genes and increase ethylene production^40^, while PpTCP.A2 can inhibit ethylene biosynthesis by negatively affecting *PpACS1* expression^41^. To broaden the transcription regulation network of ethylene biosynthesis in peach, two TFs, PpERF.A16 and PpNAC.A59 were isolated from peach fruit. Of these, PpERF.A16 could directly bind to the *PpACS1* and *PpACO1* promoters to positively regulate ethylene biosynthesis in peach fruit. *PpNAC.A59* was coexpressed with *PpERF.A16*, and PpNAC.A59 could directly bind to the promoter of *PpERF.A16* to indirectly promote ethylene biosynthesis in peach fruit. This study further elucidates the molecular network of ethylene-induced fruit ripening in peach and other perennial fruit trees.

## Results

### Identification of *ACS*, *ACO*, and *ERF* genes during fruit ripening

**Fig. 1:**
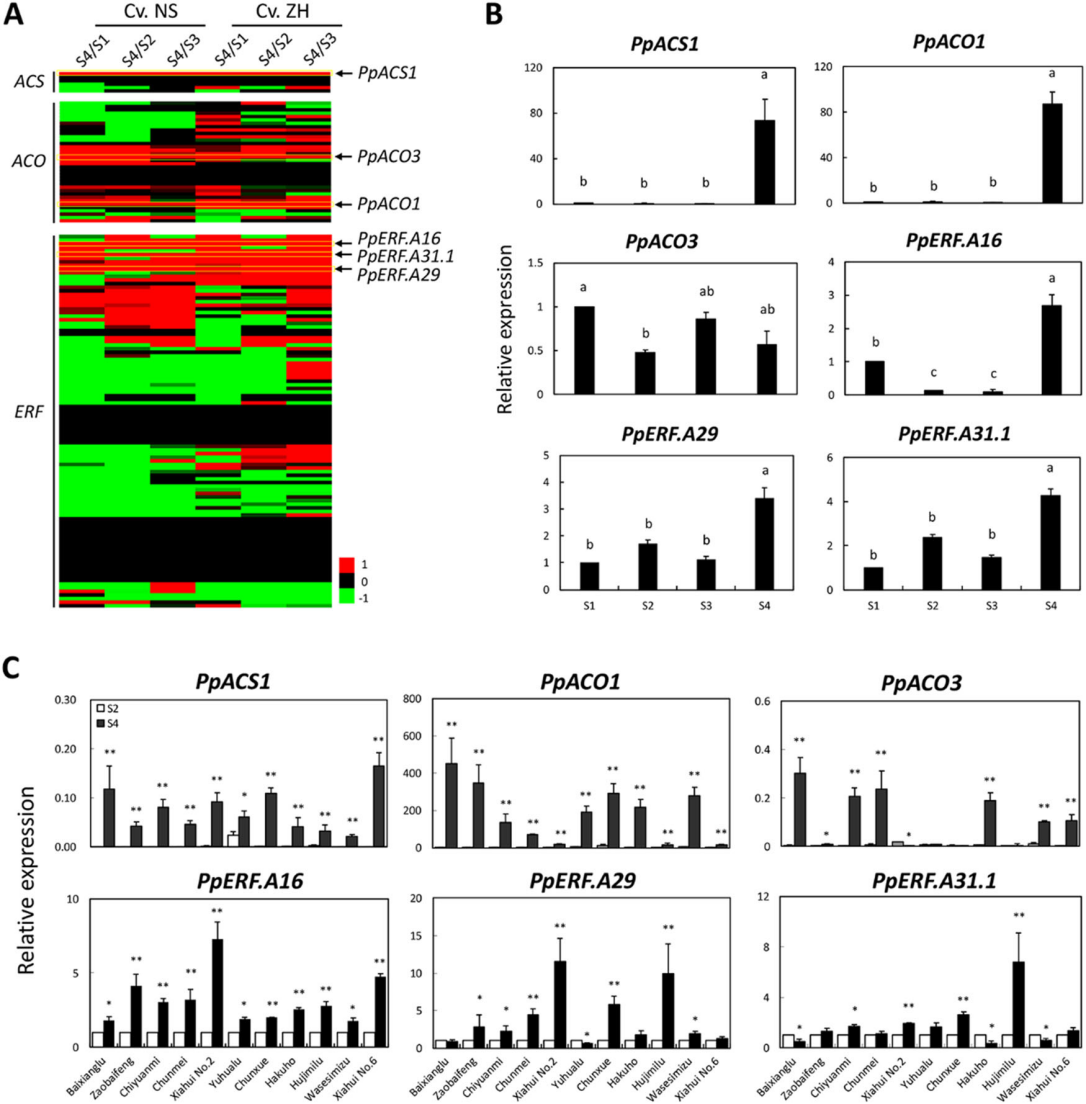
Transcriptome-based identification of the *ACS*, *ACO*, and *AP2/ERF* genes potentially associated with peach fruit ripening. **A** Transcriptome-based analysis of the differentially expressed *ACS*, *ACO*, and *AP2/ERF* genes between developing (S1 to S3) and ripening (S4) fruits in two peach cvs. NS and ZH. The color bar is calculated by log_2_ fold-change value of the ratio. **B** The expression profiles of *PpACS1*, *PpACO1*, *PpACO3*, *PpERF.A16*, *PpERF.A29*, and *PpERF.A31.1* were determined by quantitative real-time PCR (qRT-PCR) in cv. Xiahui No.5 fruits at S1, S2, S3, and S4. **C** The expression levels of *PpACS1*, *PpACO1*, *PpACO3*, *PpERF.A16*, *PpERF.A29*, and *PpERF.A31.1* were determined in the fruits of 11 peach cultivars at S2 and S4

Members of the *ACS*, *ACO*, and *ERF* gene families were isolated from peach and other fruit trees. A total of 106 *AP2/ERF* genes (Table S1) were detected in the peach genome and could be divided into two groups, A and B (Fig. S1). Of the two groups, group A corresponded to subfamily ERF, which comprised 56 clusters (A1 → A56), and group B contained the AP2, RAV, and soloist subfamilies. Moreover, six members of the *ACS* gene family were detected in peach (Fig. S2) along with 32 possible members of the *ACO* (or *ACO-like*) gene family (Fig. S3). Based on the RNA-Seq data of fruit samples of the peach cultivars Nanshantiantao (NS) and Zhaohui (ZH) in a previous study^34^, the *ACS*, *ACO*, and *AP2/ERF* genes, which had higher levels of expression in ripening fruit than in developing fruit (Fig. 1A and Table S2), were detected. Based on previous reports^37,40^, the *ACS* gene *Ppa004774m* was identical to *PpACS1*, and the two *ACO* genes, *Pp008791m* and *Ppa009228m*, were identical to *PpACO1* and *PpACO3*, respectively. The three *ERF* genes belonged to the A16, A29, and A31 clusters and were thus designated *PpERF.A16* (Ppa008730m), *PpERF.A29* (Ppa021711m), and *PpERF.A31.1* (Ppa007193m), respectively (Table S1). However, the expression level of *PpACO3* in ripening fruit was similar to that in developing fruit of cv. Xiahui No. 5 (Fig. 1B) and cvs. Yuhualu, Chumei, and Hujimilu (Fig. 1C). Moreover, *PpERF.31.1* and *PpERF.A29* had similar levels of expression between developing and ripening fruits of some cultivars (Fig. 1C), although these two genes were more highly expressed in ripening fruit than in developing fruit of cv. Xiahui No.5 (Fig. 1B). These results indicate that *PpACO3*, *PpERF.31.1*, and *PpERF.A29* are not involved in peach fruit ripening. In contrast, *PpACS1*, *PpACO1*, and *PpERF.A16* were more highly expressed in ripening fruit than in developing fruit of all 12 tested cultivars (Fig. 1B, C) and thus are probably associated with peach fruit ripening.

### Interaction of PpERF.A16 with the promoters of the *PpACS1*, *PpACO1*, and *PpendoPGM* genes

**Fig. 2:**
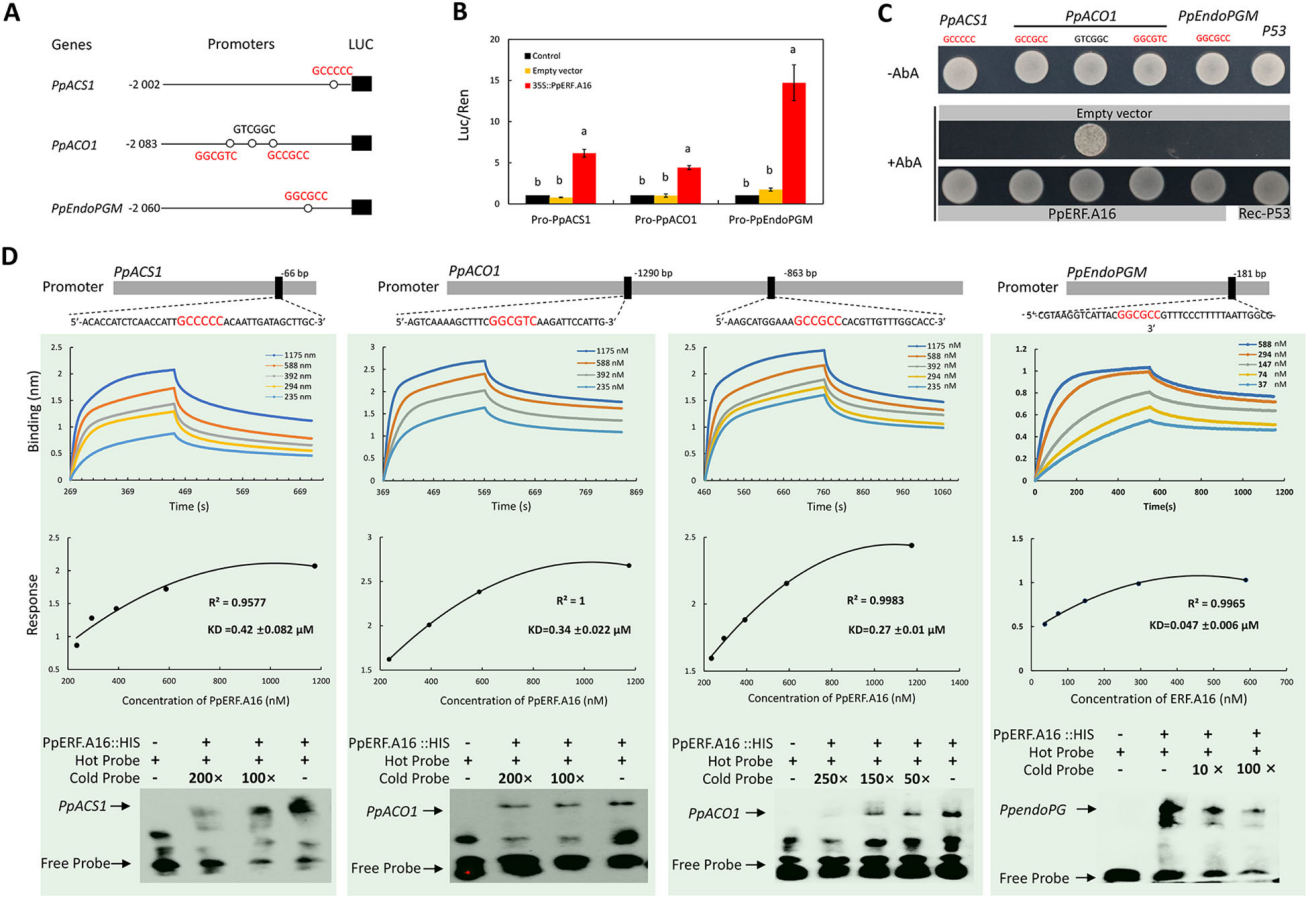
PpERF.A16 directly interacts with the promoters of *PpACS1*, *PpACO1*, and *PpendoPGM*. **A** GCC-box element(s) were predicted in the promoters of *PpACS1*, *PpACO1*, and *PpendoPGM*. **B** PpERF.A16 activated *LUC* expression that was controlled by the promoters of *PpACS1*, *PpACO1*, or *PpendoPGM*. **C** Y1H assay of PpERF.A16 with the predicted GCC-box elements in the *PpACS1, PpACO1*, and *PpendoPGM* promoters. **D** Kinetic assay and EMSA of PpERF.A16 with the *PpACS1, PpACO1*, and *PpendoPGM* promoters. + and − indicate the presence and absence of recombinant PpERF.A16::HIS, biotin-labeled probe, or cold probe, respectively. Cold probe concentrations were tenfold (10×), 50-fold (50×), 100-fold (100×), 150-fold (150×), 200-fold (200×), or 250-fold (250×) those of the labeled probes

Similar to the previous study^40^, to test whether PpERF.A16 could bind to the promoters of ripening-related genes, *PpACS1*, *PpACO1*, and *PpendoPGM*, dual-luciferase assays were conducted and revealed that the *LUC* activities that were individually driven by the *PpACS1*, *PpACO1*, or *PpendoPGM* promoters were significantly increased in *Arabidopsis* protoplasts overexpressing *PpERF.A16* (35S::PpERF.A16) compared to *Arabidopsis* protoplasts transformed with an empty vector or without any transformation (control; Fig. 2B). This result indicates that PpERF.A16 can interact with the promoters of *PpACS1*, *PpACO1*, and *PpendoPGM*. Subsequently, yeast one-hybrid (Y1H) assays showed that PpERF.A16 binds to the *PpACS1*, *PpACO1*, and *PpendoPGM* promoters (Fig. 2C). Moreover, a PpERF.A16::HIS fusion protein was generated by inserting the whole sequences of *PpERF.A16* into the pCold-TF vector. Kinetic assay showed that binding values between PpERF.A16::HIS, and the promoters of the three ripening-related genes gradually increased with increasing concentrations of PpERF.A16::HIS (Fig. 2D). Furthermore, electrophoretic mobility shift assay (EMSA) showed that PpERF.A16::HIS could bind to the probes of the *PpACS1*, *PpACO1*, and *PpendoPGM* promoters, and the binding signals weakened with increasing concentrations of the cold probe (Fig. 2D). Taken together, PpERF.A16 directly binds to the *PpACS1*, *PpACO1*, and *PpendoPGM* promoters to enhance their activities.

### Effect of PpERF.A16 on ethylene biosynthesis

**Fig. 3:**
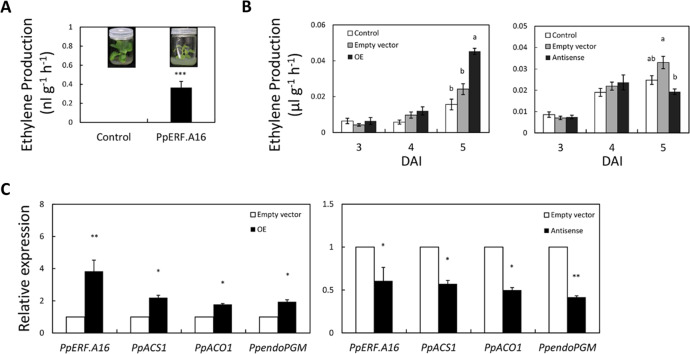
PpERF.A16 positively mediates ethylene biosynthesis. **A** The ethylene production rate was increased in tobacco transgenic plants overexpressing *PpERF.A16* compared to the wild-type control. **B** The peach fruits harvested ~10 days before the commercial harvesting date were transiently transformed with an empty vector or a vector overexpressing *PpERF.A16* (OE) or antisense *PpERF.A16* (Antisense). Ethylene production rates were measured at 3, 4, and 5 days after injection (DAI) of *Agrobacteria* containing the vectors and showed a significant increase in the OE fruits but a decrease in the antisense fruits compared to the fruit injected with the pCAMBIA1301 empty vector and noninjected control fruit at 5 DAI. **C** The expression levels of *PpERF.A16*, *PpACS1*, *PpACO1*, and *PpendoPGM* genes were increased in the OE fruits but decreased in the antisense fruits compared to the empty vector fruits at 5 DAI

To unravel the role of PpERF.A16 in ethylene biosynthesis, overexpression and antisense vectors of *PpERF.A16* were individually constructed and then introduced to *Agrobacterium tumefaciens*. After introducing the overexpression vector of *PpERF.A16* into tobacco and identifying the positive lines (Fig. S4), the ethylene production rate was measured using a gas chromatography system. The results showed that the ethylene production rate was higher in the transgenic lines of *PpERF.A16* (*P* value < 0.001) than in the nontransgenic regeneration lines (Fig. 3A), suggesting that PpERF.A16 can promote ethylene biosynthesis in transgenic tobacco. Furthermore, *Agrobacterium tumefaciens* containing overexpression and antisense vectors of *PpERF.A16* was independently injected into the fruits harvested at ~10 days before the commercial harvesting date, and ethylene production rates were measured at 3, 4, and 5 days after injection (DAI). At 5 DAI, the ethylene production rate was increased in the fruits overexpressing *PpERF.A16* but decreased in the fruits silencing *PpERF.A16* compared to the fruits infiltrated with the empty vector (Fig. 3B). Thus, PpERF.A16 can positively mediate ethylene biosynthesis during peach fruit ripening.

To test whether the expression of the *PpACS1*, *PpACO1*, and *PpendoPGM* genes was affected by PpERF.A16, qRT-PCR analyses were conducted on the transiently transformed peach fruits. As a result, both *PpACS1* and *PpACO1* were upregulated in fruits overexpressing *PpERF.A16* but downregulated in the fruits silencing *PpERF.A16* compared to the fruits infiltrated with the empty vector (Fig. 3C). Further qRT-PCR analysis showed that the transcript levels of *PpERFA15.1*/*15.2*/*15.3* and *PpERFA17.2*/*17.2* were most closely related to *PpERF.A16* and were not downregulated in the fruits with silenced *PpERF.A16* (Fig. S5). These results suggest that PpERF.A16 can positively regulate ethylene biosynthesis by inducing the expression of both *PpACS1* and *PpACO1*. In addition, PpERF.A16 upregulated the expression of *PpendoPGM* (Fig. 3C) and ripening-related genes (such as *PpCHI*, *PpPDS*, *PpZEP*, and *PpNECD*) (Fig. S6). Together with the results of the promoter analyses described above, PpERF.A16 was shown to be able to directly affect the expression of the *PpACS1*, *PpACO1*, and *PpendoPGM* genes.

### Identification of the NAC TF coexpressed with *PpERF.A16*

**Fig. 4:**
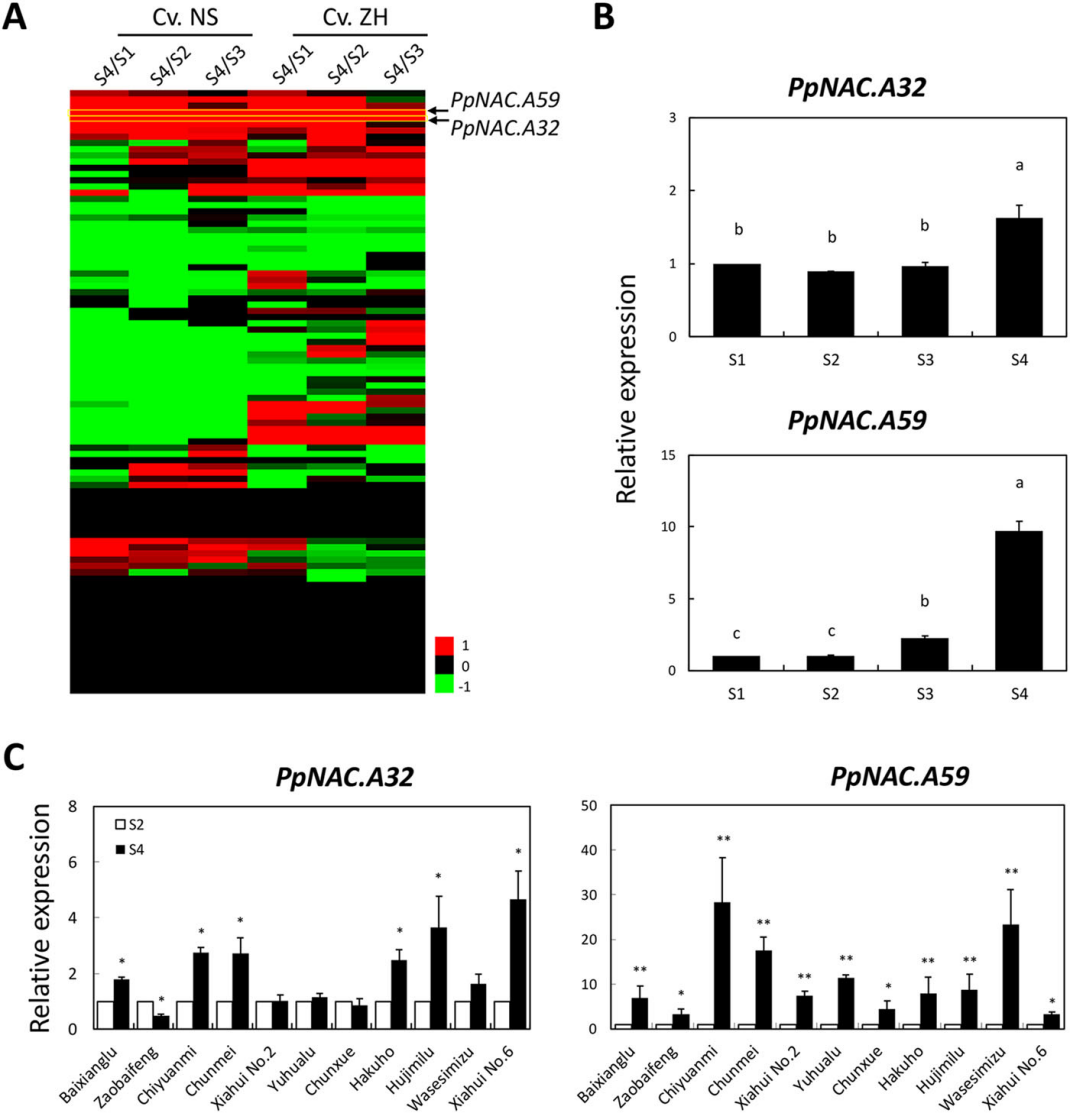
Identification of the *NAC* genes probably associated with peach fruit ripening. **A** Transcriptome analysis showed that *PpNAC.A59* and *PpNAC.A32* were differentially expressed between developing (S1 to S3) and ripening (S4) fruits in peach cvs. NS and ZH. The color bar was calculated by the log_2_ fold-change values of the ratio. **B** The expression profiles of *PpNAC.A59* and *PpNAC.A32* were tested by qRT-PCR in cv. Xiahui No. 5 fruits at S1, S2, S3, and S4. **C** The expression levels of *PpNAC.A59* and *PpNAC.A32* were tested in the fruits of 11 peach cultivars at S2 and S4

To mine the upstream TF(s) of *PpERF.A16* expression and ethylene biosynthesis, the potential *cis*-regulatory elements in the *PpERF.A16* promoter were predicted, revealing 12 elements (the sequences with red color) for the NAC TF (Fig. S7). A higher number of elements for the NAC TF indicated that this TF should play an important role in mediating *PpERF.A16* expression and ethylene production. Subsequently, 103 members of the *NAC* gene family were isolated from the peach genome. Together with the *NAC* genes derived from other fruit trees, the *NAC* gene family can be divided into two groups, A and B (Fig. S8 and Table S3). Of the two groups, group A comprised 69 clusters (A1 → A69), while group B contained only three clusters (B1 → B3; Fig. S8). Based on the RNA-Seq data of fruit samples of cvs. NS and ZH in a previous study^34^, two *NAC* genes were more highly expressed in ripening fruit than in developing fruit of both cvs. NS and ZH (Fig. 4A and Table S2). These two genes, *Ppa007445m* and *Ppa008828m*, belong to clusters A32 and A59 and were thus designated *PpNAC.A32* and *PpNAC.A59*, respectively (Table S3). Of the two genes, however, *PpNAC.A32* had similar levels of expression between developing and ripening fruits of cv. Xiahui No.5 (Fig. 4B) and cvs. Xiahui No. 2, Yuhualu, Chunxue, and Wasesimizu (Fig. 4C), while *PpNAC.A59* was more highly expressed in ripening fruit than in developing fruit of all 12 tested cultivars (Fig. 4B, C). Obviously, *PpNAC.A59* had an identical expression profile to *PpERF.A16* in all tested peach cultivars, indicating that this TF may induce the expression of *PpERF.A16* and is probably associated with peach fruit ripening.

### Interaction of PpNAC.A59 with the *PpERF.A16* promoter

**Fig. 5:**
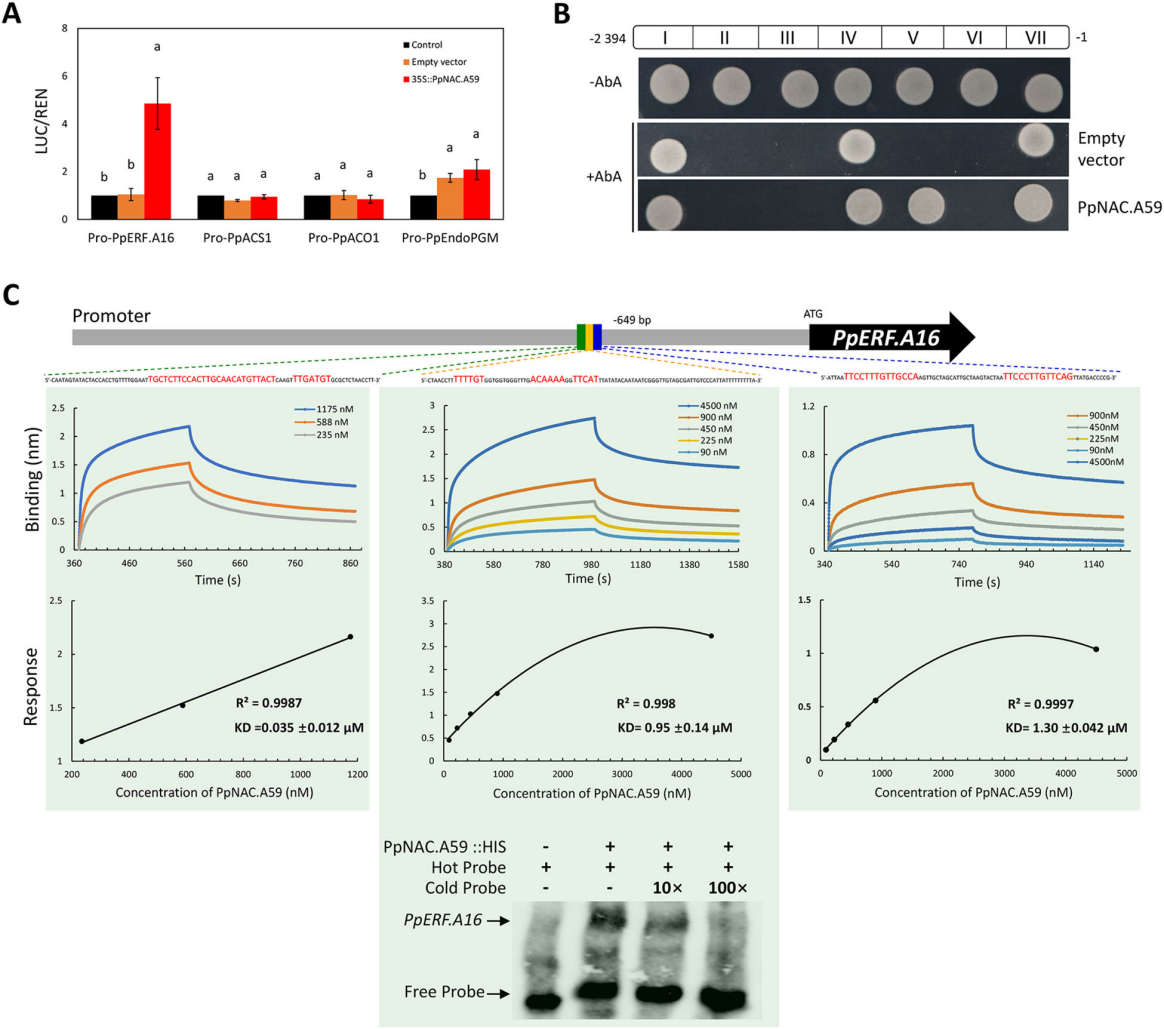
PpNAC.A59 directly interacts with the *PpERF.A16* promoter. **A** PpNAC.A59-activated *LUC* expression, which was controlled by *PpERF.A16* promoter. **B** Y1H assay of PpNAC.A59 with seven fragments (I to VII) in the *PpERF.A16* promoter. **C** Kinetic assay and EMSA of PpNAC.A59 with the *PpERF.A16* promoter. “+” and “−” indicate the presence and absence of recombinant PpNAC.A59::HIS, biotin-labeled probe, or cold probe, respectively. Cold probe concentrations were tenfold (10×) and 100-fold (100×) those of the labeled probes

To test whether PpNAC.A59 interacts with the *PpERF.A16* promoter, a dual-luciferase assay was conducted and showed an interesting result. The *LUC* gene driven by the *PpERF.A16* promoter had higher activity in *Arabidopsis* protoplasts overexpressing *PpNAC.A59* (35S::PpNAC.A59) than in *Arabidopsis* protoplasts transformed with an empty vector or without any transformation (control; Fig. 5A). In contrast, the *LUC* gene driven by any promoter of *PpACS1*, *PpACO1*, and *PpendoPGM* had similar activity in *Arabidopsis* protoplasts overexpressing *PpNAC.A59* to that in *Arabidopsis* protoplasts transformed with an empty vector (Fig. 5A). Therefore, PpNAC.A59 interacts with the *PpERF.A16* promoter.

To determine which fragment(s) in the promoter of *PpERF.A16* could be bound by PpNAC.A59, the promoter sequences of *PpERF.A16* were fragmented into seven sections (I → VII). Only fragment V was bound by PpNAC.A59 in yeast cells (Fig. 5B). In fragment V, we detected two possible *cis*-regulatory elements (WNNYBTNNNNNNNAMGNHW and TTRCGT) for NAC TF^42–44^, and these elements were used to synthesize the biotin-labeled probes (Fig. 5C). Subsequently, a recombinant PpNAC.A59::HIS fusion protein was generated by inserting the whole sequences of *PpNAC.A59* into the pCold-TF vector. Kinetic assay showed that the binding value between PpNAC.A59::HIS and each of the three probes gradually increased with increasing concentrations of PpNAC.A59::HIS, and the binding response between PpNAC.A59::HIS and the probe boxed with a yellow color peaked when the concentration reached 4500 nM (KD <1 μM; Fig. 5C). Moreover, EMSA showed that PpNAC.A59::HIS could bind to the probe boxed with a yellow color, and the binding signal weakened as the concentration of the cold probe increased (Fig. 5C). Taken together, PpNAC.A59 can directly bind to the promoter of *PpERF.A16*.

### Effect of PpNAC.A59 on ethylene biosynthesis

**Fig. 6:**
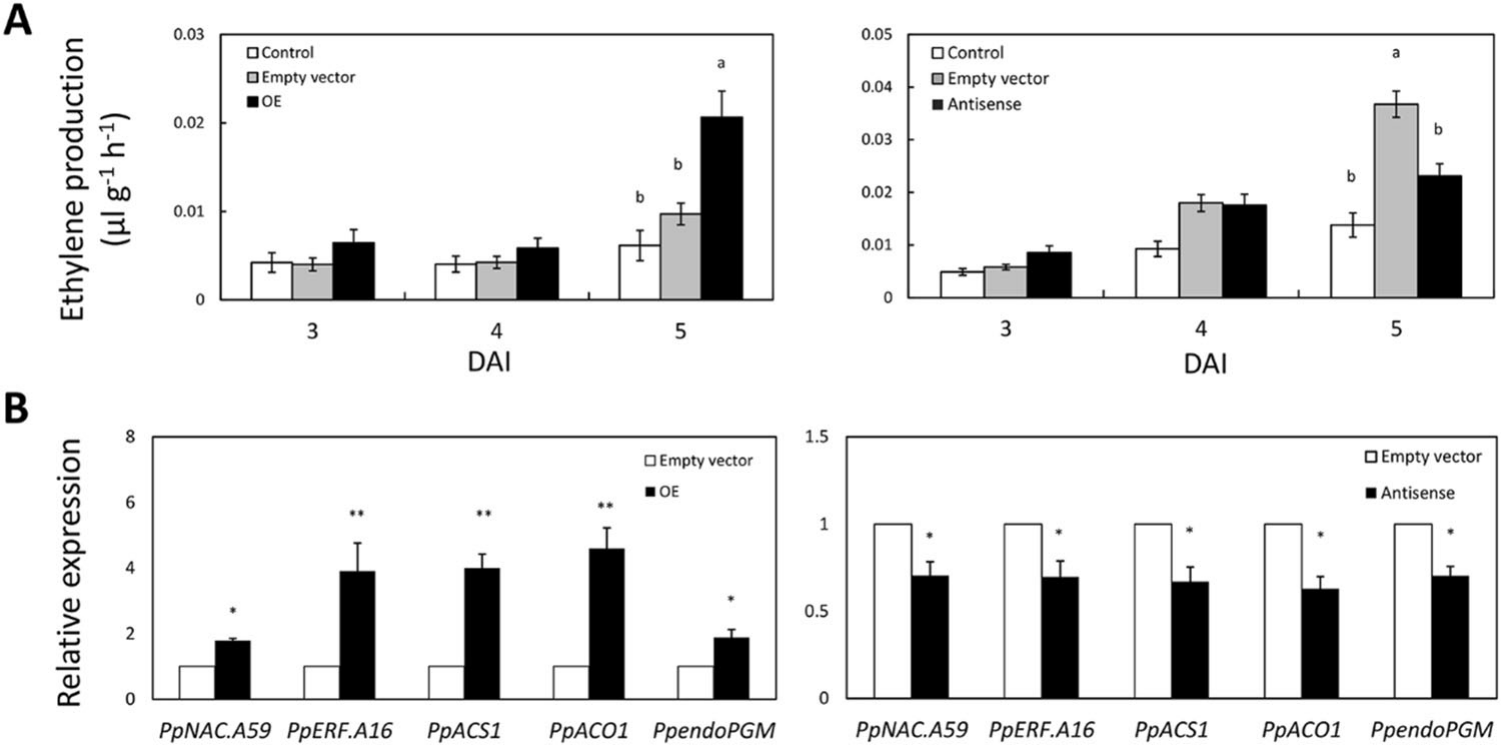
PpNAC.A59 positively mediates ethylene biosynthesis. **A** The peach fruits harvested ~10 days before the commercial harvesting date were transiently transformed with an empty vector or a vector overexpressing *PpNAC.A59* (OE) or antisense *PpNAC.A59* (Antisense). Ethylene production rates were measured at 3, 4, and 5 days after injection (DAI) of *Agrobacteria* containing the vectors and showed a significant increase in the OE fruits but a decrease in the antisense fruits compared to the fruits injected with the empty vector pCAMBIA1301 and noninjected control fruits at 5 DAI. **B** The expression levels of *PpERF.A16*, *PpACS1*, *PpACO1*, and *PpendoPGM* genes were increased in the OE fruits but decreased in the antisense fruits compared to the empty vector fruits at 5 DAI

To unravel the role of PpNAC.A59 in ethylene biosynthesis, overexpression and antisense vectors of *PpNAC.A59* were constructed and then introduced into *A. tumefaciens*. The resuspended *Agrobacterium* was injected into peach fruits, and ethylene production rates were measured at 3, 4, and 5 DAI. At 5 DAI, the ethylene production rate was increased in the fruits overexpressing *PpNAC.A59* but reduced in the fruits with silenced *PpNAC.A59* compared to the fruits injected with an empty vector (Fig. 6A). This result indicated that PpNAC.A59 can positively regulate ethylene biosynthesis during peach fruit ripening.

To test whether the expression of ethylene biosynthesis and ripening-related genes was affected by PpNAC.A59, the transiently transformed peach fruits were analyzed using qRT-PCR. The results showed that the expression levels of *PpACS1*, *PpACO1*, and *PpERF.A16* were upregulated in fruits overexpressing *PpNAC.A59* and downregulated in the fruits with silenced *PpNAC.A59* compared to the fruits infiltrated with the empty vector (Fig. 6B). Further qRT-PCR analysis showed that the transcript levels of *PpNAC.A58/A50/A61*, which were most closely related to *PpNAC.A59*, were not downregulated in the fruits with silenced *PpNAC.A59* (Fig. S9). In addition, PpNAC.A59 upregulated the expression of *PpendoPGM* (Fig. 6B) and other ripening-related genes (such as *PpCHI*, *PpZEP*, and *PpNECD)* (Fig. S10). Due to no interaction being detected between PpNAC.A59 and the promoter sequences of *PpACS1*, *PpACO1*, and *PpendoPGM* (Fig. 5A), it is likely that PpNAC.A59 indirectly promotes the transcription of these genes via the PpERF.A16-ethylene pathway.

## Discussion

### PpERF.A16 directly regulates ethylene biosynthesis during fruit ripening

AP2/ERF TFs have been characterized in plant species, including peach^45,46^, and are involved in various biological processes, such as plant growth, development, senescence, and stress tolerance^6,47^. In fleshy fruits, AP2/ERF TFs play important roles in transmitting the ethylene signal and stimulating activities of downstream genes responsive to fruit ripening^6,19^. This type of *AP2/ERF* gene has been identified in climacteric fruits such as apple, banana, and pear^11–13^. This study identified a similar gene *PpERF.A16* from previously reported RNA-Seq data from peach fruit. Overexpression of *PpERF.A16* could enhance the expression of *PpACS1* and *PpACO1* and thus increase ethylene production during peach fruit ripening (Fig. 3). Furthermore, PpERF.A16 could also induce the expression of other ripening-related genes (Figs. 3C, S6) and is thus likely associated with peach fruit ripening. *PpERF.A16* showed a similar function to the previously reported *ERF* genes *MdERF3*, *PbERF24*, and *MaERF9* involved in regulating the ripening of climacteric fruits^11–13^. However, a difference is that PpERF.A16 binds to the promoters of both the *PpACS1* and *PpACO1* genes (Fig. 2), while MdERF3, PbERF24, and MaERF9 interact with the promoters of an *ACS* or *ACO* gene but not both genes^11–13^. Another difference is that these genes are clustered into different subgroups of the ERF family, i.e., PpERF.A16, PbERF24, MdERF3, and MaERF9 were clustered into the A16, A17, A39 and A13 subgroups, respectively (Table S1).

It has been reported that exogenous auxin can effectively induce the expression of *PpACS1* and *PpERF.A16*^37,48^. Ethylene production is associated with the TC microsatellite genotypes of *PpYUC11* in peach fruits with flesh melting and stony hard phenotypes^49,50^. Recently, it was confirmed that auxin can promote ethylene biosynthesis in apple fruit by activating MdARF5^16^. MdARF5 can bind to the *MdACS1*, *MdACS3a*, *MdACO1*, and *MdERF2* promoters to induce their expression in apple fruit. It is possible that auxin induces ethylene biosynthesis in peach fruit by an ARF TF. However, exogenous auxin treatment cannot upregulate ARF TFs in peach fruit^37,48^. Instead, the expression levels of *auxin/indole-3-acetic acid* (*auxin/IAA*) genes (*IAA11*, *IAA13*, and *IAA9_2*) were increased in the peach fruits with exogenous auxin treatment compared with those in the control fruits^37,48^. It is more possible that these auxin/IAA TFs directly or indirectly induce the expression of *PpACS1* and *PpERF.A16* to promote ethylene biosynthesis in peach fruit.

### PpNAC.A59 indirectly mediates ethylene biosynthesis during fruit ripening

Plant-specific NAC TFs of woody and grassy plants have been phylogenetically analyzed^51,52^ and are reported to regulate various physiological traits^53–56^. *NOR* was the first *NAC* gene known to promote ethylene biosynthesis in a nonripening tomato mutant^22^. Subsequently, the *SlNAC4* and *NOR-like 1* genes were proven to be associated with fruit ripening in tomato^21,23^, similar to *AdNAC6* and *AdNAC7* in kiwifruit^25^. In the present study, *PpNAC.A59* was shown to have a higher level of expression in ripening fruit than in developing fruit of all tested peach cultivars (Fig. 4). Interestingly, PpNAC.A59 cannot interact with the promoters of *PpACS1* and *PpACO1* (Fig. 5A) but could induce the expression of both *PpACS1* and *PpACO1* to promote ethylene biosynthesis in transiently transformed fruits (Fig. 6). This result is different from previous studies of the NACs associated with ethylene biosynthesis during fruit ripening. For example, NOR-like 1 of tomato interacts with the *SlACS2* and *SlACS4* promoters^21^, and AdNAC6/7 of kiwifruit interacts with the *AdACS1* and *AdACO1* promoters^25^. In contrast, PpNAC.A59 directly interacts with the *PpERF.A16* promoter (Fig. 5) but not with the *PpACS1* or *PpACO1* promoter. Then, PpERF.A16 interacts with the promoters of *PpACS1* and *PpACO1*. Obviously, PpNAC.A59 indirectly mediates ethylene biosynthesis during peach fruit ripening. It is noteworthy that PpNAC.A59 was not clustered together with any of the previously reported NACs associated with ethylene biosynthesis during fruit ripening. PpNAC.A59, AdNAC6/7, NOR/NOR-like 1, SlNAC1, and SlNAC4 were individually clustered into subgroups A59, A21, A35, A25, and A24 of the NAC family, respectively (Table S3).

In previous studies, NACs were reported to be involved in abscisic acid (ABA) biosynthesis^57,58^. Both *Oryza sativa* OsNAC2 and *Solanum lycopersicum* SlNAP2 directly control the expression of the ABA biosynthesis gene *9-cis-epoxycarotenoid dioxygenase* and the ABA catabolic gene *ABA 8’-hydroxylase* to affect ABA production^59,60^. It is noteworthy that ABA indirectly promotes fruit ripening by triggering ethylene biosynthesis in climacteric fruits^61,62^. In this study, we found that PpNAC.A59 positively regulated the expression of ABA biosynthesis genes (*Zeaxanthin epoxidase* and *9-cis-epoxycarotenoid dioxygenase*; Fig. S10), indicating that PpNAC.A59 is likely involved in ABA biosynthesis in peach fruit. Therefore, ethylene biosynthesis may also be influenced by the PpNAC.A59-ABA-ethylene signal cascade during peach fruit ripening.

### Molecular network of fruit ripening in peach

Controlling the ripening process of climacteric fruits is important to the commercialization of these fruit crops and has been widely studied at the molecular genetics level^19,63^. In tomato, a genetic network regulating fruit ripening has been established and consists of TFs of different families, including RIN, FUL1, FUL2, HB-1, AP2a, ERF6, TAGL1, NOR, CNR, and TDR4^29,30^. This network was further enriched by the most recently identified TFs, SlNAC4, SlNAC1, SlZFP2, and NOR-like 1^20,21,23,24^, and epigenetic modifiers, such as DNA methylation^64,65^, microRNA^66,67^, and long noncoding RNA^68,69^. Obviously, the understanding of the genetic network regulating fruit ripening in tomato is much advanced compared to our understanding of the network in tree fruits. During apple fruit ripening, the regulatory network of ethylene biosynthesis was constructed to include the TFs MYC2, ARF5, ERF2, and ERF3 and ethylene biosynthesis genes^12,15,16^. However, in other perennial fruit trees, few TFs were concatenated into a network. In peach fruit, two TFs, PpHB.G7 and PpTCP.A2, were identified in the previous studies^40,41^. Of these two TFs, the transcription activator PpHB.G7 can directly bind to the *PpACS1* and *PpACO1* promoters to increase ethylene production^40^, while the transcription repressor PpTCP.A2 can inhibit the expression of *PpACS1* to hinder ethylene biosynthesis^41^.

**Fig. 7:**
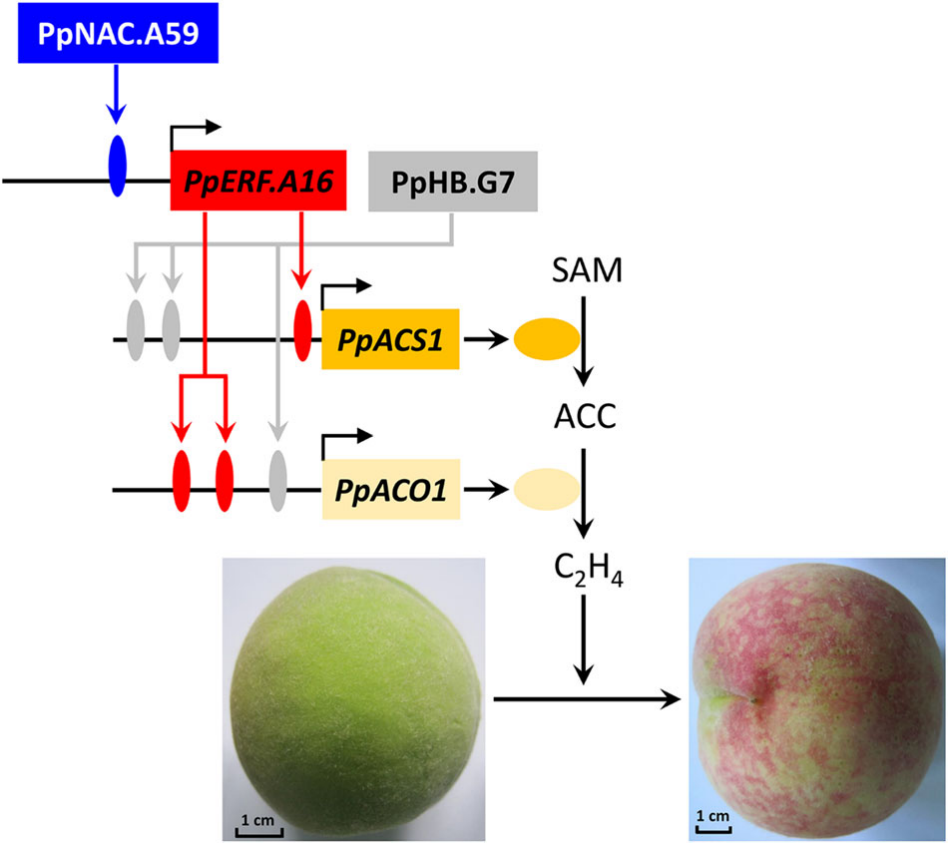
A regulatory model of ethylene biosynthesis induced by PpNAC.A59-activated *PpERF.A16*. PpNAC.A59 activates the expression of *PpERF.A16* by directly binding to its promoter. PpERF.A16 and PpHB.G7 enhance the expression of both *PpACS1* and *PpACO1* by directly interacting with their promoters, resulting in an increase in ethylene (C_2_H_2_) production in peach fruit

In the present study, two TFs, PpERF.A16 and PpNAC.A59, were shown to be involved in ethylene biosynthesis during peach fruit ripening. These results could be integrated into a primary network of fruit ripening in peach (Fig. 7). First, PpNAC.A59 can induce the expression of *PpERF.A16* by directly binding to its promoter. Second, PpERF.A16 and PpHB.G7 enhances the expression of both *PpACS1* and *PpACO1* by directly interacting with their promoters, contributing to an increase in ethylene production. Finally, ethylene accelerates fruit ripening by mediating fruit metabolism, such as flavonoid and carotenoid metabolism. It is noteworthy that this conclusion is supported by a previous study in which the NAC TF was predicted to control ethylene biosynthesis during peach fruit ripening^70^. The only difference is that the predicted NAC TF can induce the expression of ethylene biosynthesis genes by directly interacting with their promoters^70^, while PpNAC.A59 indirectly induces the expression of both *PpACS1* and *PpACO1* by directly mediating the expression of *PpERF.A16*. Further work will focus on the enrichment of this network by identifying more TFs associated with fruit ripening.

## Materials and methods

### Plant materials

A total of 12 peach cultivars were used in this study. Of these cultivars, Wasesimizu and Xiahui No. 5 were maintained at the Jiangsu Academy of Agricultural Sciences (Nanjing, Jiangsu Province, China). The other ten cultivars (Chunxue, Hakuho, Hujinmilu, Xiahui No. 2, Yuhualu, Chiyuanmi, Chunmei, Xiahui No. 6, Baixianglu, and Zaobaifeng) were identical to those in a previous study^40^. The cv. Baixianglu fruit presents a semifreestone melting flesh phenotype, and the fruits of the other ten peach cultivars present a clingstone melting flesh phenotype. Based on the dynamic investigation of fruit development and ripening^71^, the fruits of cv. Xiahui No. 5 were collected at the fruitlet (S1), stone hardening (S2), preripening (S3), and ripening (S4) stages, and the fruits of the other 11 cultivars were collected at the rapid enlargement (roughly corresponding to S2) and ripening stages (S4; Table S4). Grouping, biological replication, and storage of fruit samples were as described in a previous study^40^.

### Sequence and phylogenetic analyses of the *ACS*, *ACO*, *AP2/ERF*, and *NAC* genes

To isolate the *ACS*, *ACO*, *AP2/ERF*, and *NAC* genes in peach and other fruit trees, the nucleotide and amino acid sequences of the *ACS*, *ACO*, *AP2/ERF*, and *NAC* genes in peach were extracted from the peach genome version 1.0 (Genome Database for Rosaceae; http://www.rosaceae.org/). Then, these downloaded sequences were used as indexes to find their homologs in papaya (*Carica papaya* ASGPBv0.4), strawberry (*Fragaria vesca* v 1.1), orange (*Citrus sinensis* v1.1), grape (*Vitis vinifera* Genoscope.12X), apple (*Malus domestica* v1.0) (https://phytozome.jgi.doe.gov/), and pear (*Pyrus bretschneideri*; http://peargenome.njau.edu.cn/) genomes (Tables S1, S3). Sequence alignment and phylogenetic analyses were as described in a previous study^40^.

### Gene expression analysis in developing and ripening fruits

In a previous study, RNA-Seq was performed in the developing and ripening fruits of three peach cultivars, Nanshantiantao (NS), Zhaohui (ZH), and Myojo^34^. Of these three cultivars, NS and ZH fruits presented a melting flesh phenotype, and the fruit hardness gradually decreased during ripening, while fruits of the Myojo cultivar did not soften during fruit ripening. For this reason, the transcriptome data fruits from cvs. NS and ZH were used to isolate the *ACS*, *ACO*, *AP2/ERF*, and *NAC* genes that may be associated with fruit ripening. The hierarchical clustering of transcriptome data was performed according to a previous study^40^. By comparing the gene expression levels of the ripening fruit (S4) to those of the developing fruits (S1 to S3), we isolated the target genes *PpACS1*, *PpACO1*, *PpACO3*, *PpERF.A16*, *PpERF.A29*, *PpERF.A31.1*, *PpNAC.A32*, and *PpNAC.A59*. Thus, these eight genes were further tested by qRT-PCR analysis. The details for qRT-PCR and data analysis were identical to those of a previous study^40^.

### Transformations in peach fruit and tobacco

The full length CDSs of *PpERF.A16* and *PpNAC.A59* were amplified with the PCR primers listed in Table S5 and inserted in a sense orientation downstream of the CaMV 35S promoter in the pCAMBIA1301 vector to generate gene overexpression vectors. An approximately 400-bp fragment of the CDS of *PpERF.A16* or *PpNAC.A59* was amplified using the primers listed in Table S5 and inserted in an antisense orientation downstream of the CaMV 35 S promoter in the pSAK277 vector to create a gene silencing construct. The 400 bp fragments were located in the nonconserved region and showed no significant similarity (defined as no >21 bp in length with 100% identity) to the sequences of the most closely related genes within the same gene family (Figs. S11–S20). pCAMBIA1301 contains a hygromycin resistance gene, and pSAK277 contains a kanamycin resistance gene as a selection marker in plant transformation. These vectors were transferred into *Agrobacterium tumefaciens* EHA 105 cells that were used to transiently transform peach fruit tissues with a previously reported *Agrobacterium* infiltration protocol^40^. The fruit used for the transformation experiments was harvested from the peach cultivar Xiahui No. 5 at ~10 days before the commercial harvesting date. At three, four and five DAI, at least five fruits were sealed in a 5-L airtight jar, and six jars were used for each construct. Three DAI, ethylene production was measured every day. Fruit flesh tissues were sampled at 5 DAI for qRT-PCR analysis. All of the PCR primer sequences are listed in Supplementary Table S5.

Overexpression vectors of *PpERF.A16* were also introduced into tobacco (*Nicotiana tabacum*) based on *Agrobacterium*-mediated transformation as described previously^72^. The regenerated tobacco plants were tested by PCR for amplification of the *hygromycin* gene from the pCAMBIA1301 vector. PCR-positive plants and wild-type control plants were used for measuring ethylene production.

### Ethylene measurements

The ethylene production rate was measured using a gas chromatography system (GC2010, Shimadzu, Japan) with a 1000 μl rheodyne injector (Gaoge, Shangshai, China) according to a previously reported procotol^73^ with a few modifications. In brief, at room temperature, at least four fruits were weighed as a replicate and then placed into a 3-l airtight chamber. For tobacco, each plant was a replicate and individually weighed after collection from tissue culture medium and placed into a 15 ml centrifuge tube. Six replicates were used for each gene construct and each control. A 0.5-ml gas sample was collected from the chambers and tubes and injected into a gas chromatograph fitted with a 30-cm glass column (3.2 mm ID). Gas flows for N2, H2, and air were 40, 40, and 500 ml/min, respectively. The oven, injector, and FID temperatures were 300, 60, and 260 °C, respectively.

### Protoplast isolation and dual-luciferase assay

Protoplast isolation was as described in a previous report^40^. Approximately 2000-bp sequences upstream of the initiation codons of *PpACS1*, *PpACO1*, *PpendoPGM*, and *PpERF.A16* were inserted into a pGreenII 0800-LUC vector for a dual-luciferase assay that was performed according to a previous study^40^. At least six biological replicates were used in each assay. All of the primer sequences are listed in Table S5.

### Yeast one-hybrid assay (Y1H)

It is well known that ERF TFs can bind to the GCC-box element^74,75^. Thus, the possible GCC-box elements were surveyed from the promoters of *PpACS1*, *PpACO1*, and *PpEndoPGM* (Fig. 2A). The fragments containing the possible GCC-box element were amplified from the genomic DNA of cv. Xiahui No.5 and then inserted into the pAbAi vector. The whole sequences of *PpERF.A16* and *PpNAC.A59* genes were individually inserted into the pGADT7 vector. The Y1H assay was performed using the Matchmaker Gold Yeast One-Hybrid Library Screening System (Clontech, Palo Alto, CA). All of the primer sequences are listed in Table S5.

### Recombinant proteins of both PpERF.A16 and PpNAC.A59 in *Escherichia coli*

The whole sequences of *PpERF.A16* and *PpNAC.A59* were individually inserted into the pCold-TF expression vector (including a HIS tag) to create recombinant proteins. Recombinant plasmids were introduced into *Escherichia coli* cells and incubated at 37 °C until an OD_660_ of 0.6 was reached. Protein expression was induced at 16 °C for 24 h, and the recombinant protein was extracted and purified at 4 °C^76^. SDS-PAGE was conducted to analyze the extracted proteins. All of the primer sequences are listed in Table S5.

### Kinetic assay

Fragments containing possible protein-binding elements were used to synthesize probes biotinylated at the ends of both the N- and C-termini (Sangon, Shanghai, China). Protein-DNA interactions were determined by biolayer interferometry using a ForteBio Octet 96 Red Instrument (Menlo Park, CA, USA). The kinetic assay comprised the following five steps: (1) baseline acquisition was conducted by loading PBS buffer (1.4 mM KH_2_PO_4_, 2.7 mM KCl, 4.3 mM Na_2_HPO_4_, 137 mM NaCl, 0.02% (w/v) Tween-20; pH = 7.2) onto streptavidin-coated biosensors, (2) the biotin-labeled probe was loaded onto the sensor, (3) a second baseline acquisition was conducted with the same PBS buffer, and steps (4) and (5) were the respective association and dissociation between the recombinant protein and the biotin-labeled probe(s). Equilibrium binding affinities (KD) were measured using nanomole quantities of samples as well as rates of association and dissociation (KA/KD)^77^.

### Electrophoretic mobility shift assay (EMSA)

The recombinant proteins and biotin-labeled probes were also used for EMSAs. In brief, the binding reaction was performed in a 0.2 ml centrifuge tube for 20 min at room temperature, and the reaction mixtures contained 2 μl loading buffer, 1 mg poly(dI·dC), 5 mM MgCl_2_, 2.5% glycerol, 0.05% NP-40, 200 nM probe, and 1 μg recombinant protein, with ultrapure water added for a total volume of 20 μl. For probe competition, 2, 10, 20, 30, 40, or 50 μM nonlabeled probe was individually mixed into the reaction mixtures. After running in a 6% polyacrylamide gel, the protein-DNA complexes were electrophoretically transferred to a nylon membrane (GE Healthcare, Danderyd, Sweden). SDS-PAGE imaging was performed by a Tanon 5200 chemiluminescence imaging system (Tanon, Shanghai, China) and scanned using a ChemiDoc™ XRS+ System (BioRad, Hercules, CA).

### Statistical analysis

Means and standard errors were calculated from at least three biological replicates for qRT-PCR analysis, five biological replicates for ethylene measurement in tobacco, and at least six replicates for ethylene measurement in peach fruit. Analysis of variance was calculated by Student’s *t* test. The lowercase letters a, b, and c indicate the level of significance at *P* < 0.05. Single, double and triple asterisks represent the levels of significance at *P* < 0.05, <0.01, and <0.001, respectively.

## Supplementary information

Supplementary figures

Supplementary tables
