# Supplementary material for: The NAM/ATAF1/2/CUC2 transcription factor PpNAC.A59 enhances PpERF.A16 expression to promote ethylene biosynthesis during peach fruit ripening
Source: Hortic Res. 2021 Oct 1;8:209. doi: 10.1038/s41438-021-00644-6 (PMC8484547; doi:10.1038/s41438-021-00644-6)
Supplement: Supplementary file 1 — Supplementary figures [file 41438_2021_644_MOESM1_ESM.docx]

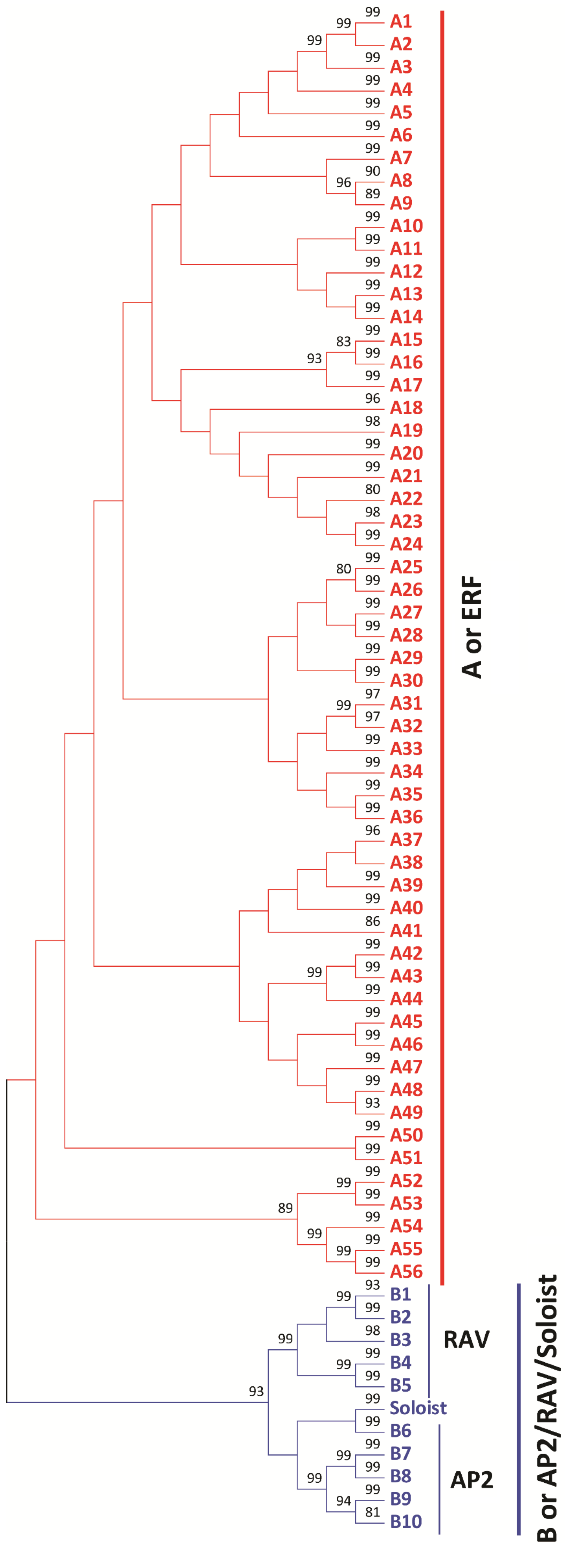


**Figure S1** Phylogenetic classification of *AP2/ERF* genes in peach and other fruit trees. Accession numbers of these genes are listed in Table S1.


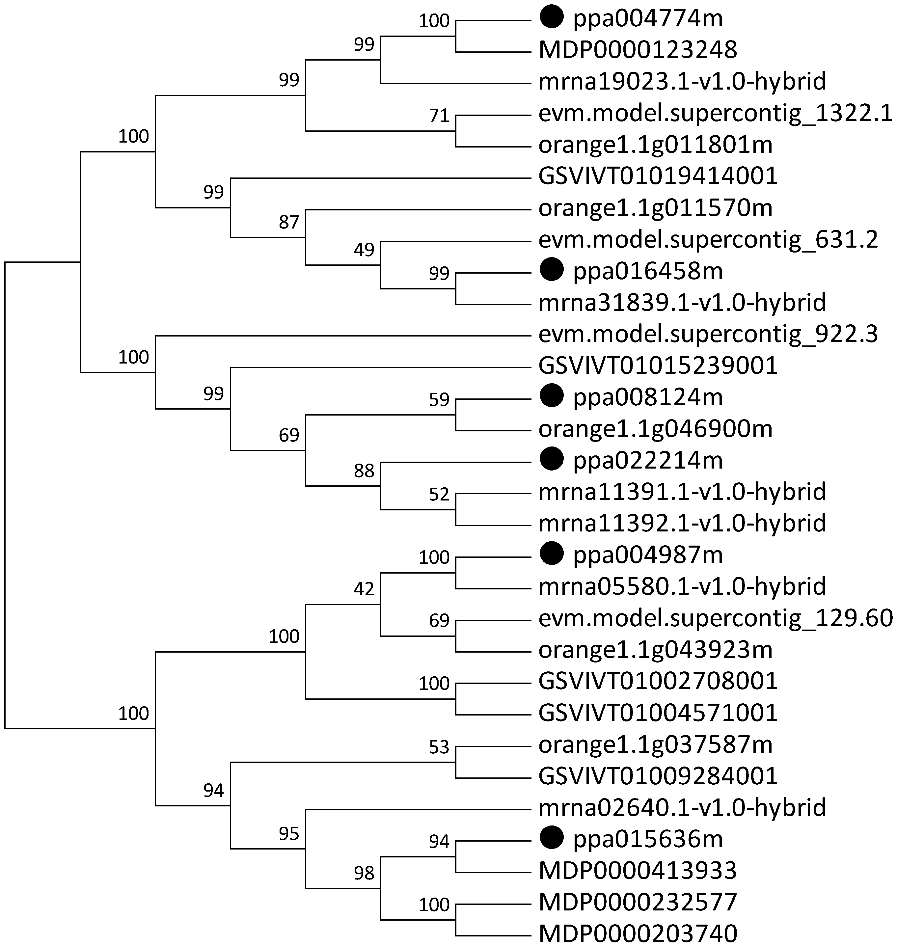


**Figure S2** Phylogenetic classification of *ACS* genes in peach and other fruit trees.


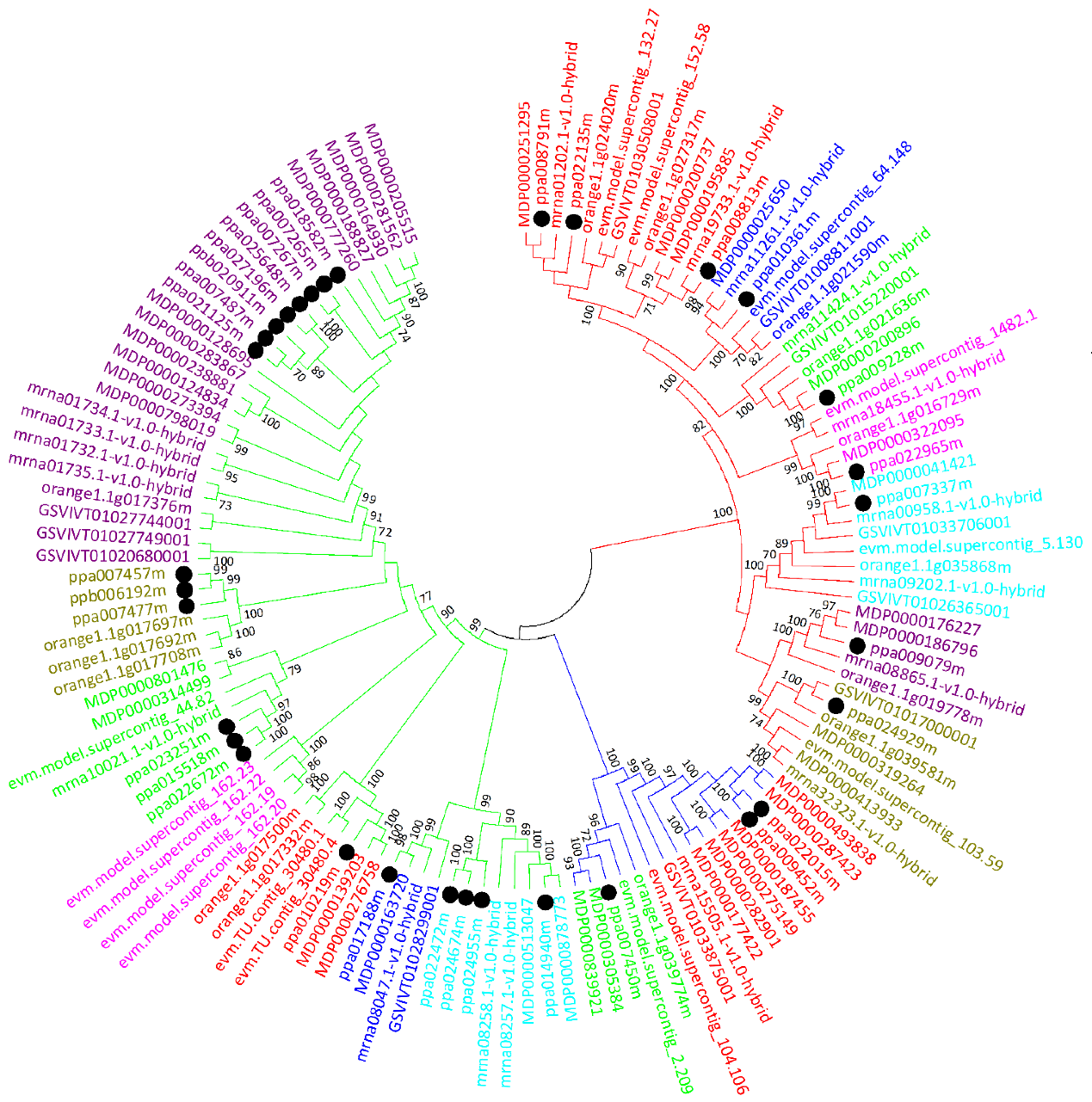


**Figure S3** Phylogenetic classification of *ACO* genes in peach and other fruit trees.


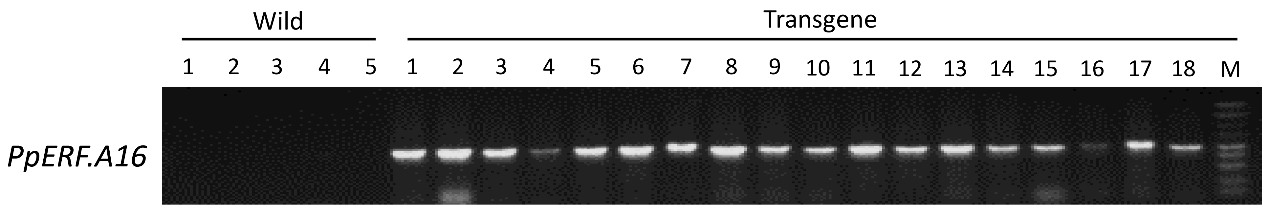


**Figure S4** Identification of positive transgenic lines of *PpERF.A16* in tobacco. The re-generation tobacco plants of PpERF.A16 were tested by PCR using the primer pairs designed from *Hygromycin* gene in the pCAMBIA1301 vector.


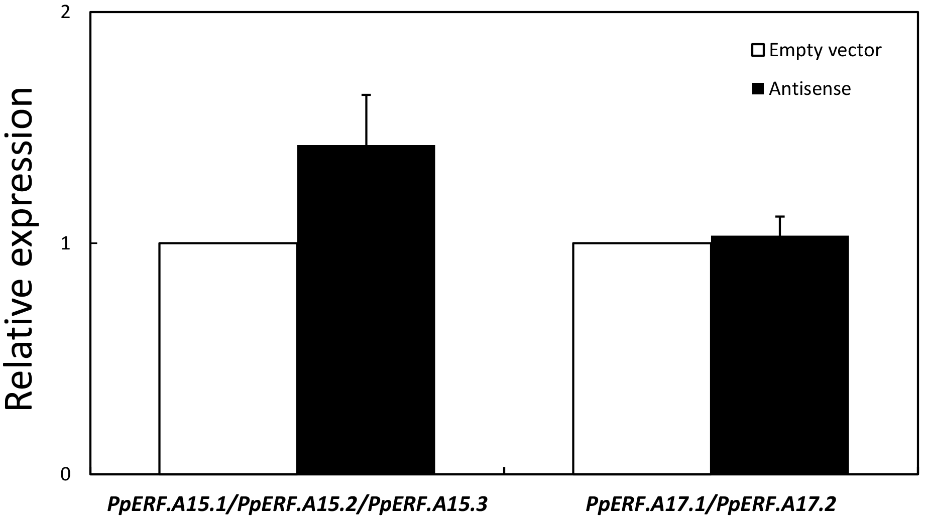


**Figure S5** Expression level of genes closely related to *PpERF.A16* in the fruit silencing *PpERF.A16*. Due to the extremely high bootstrap value (93) among A15, A16, and A17 (Figure S1), the members of these three group in peach were tested in the fruit silencing *PpERF.A16*. However, the nucleotide acid sequences of *PpERF.A15.1*, *PpERF.A15.2*, and *PpERF.15.3* were almost identical and thus shared a primer pair (Table S5). Similarly, the nucleotide acid sequences of *PpERF.A17.1* and *PpERF.A17.2* were almost identical and thus also shared a primer pair (Table S5). The result showed that these genes were not differentially expressed in the fruit silencing PpERF.A16 compared to the control.


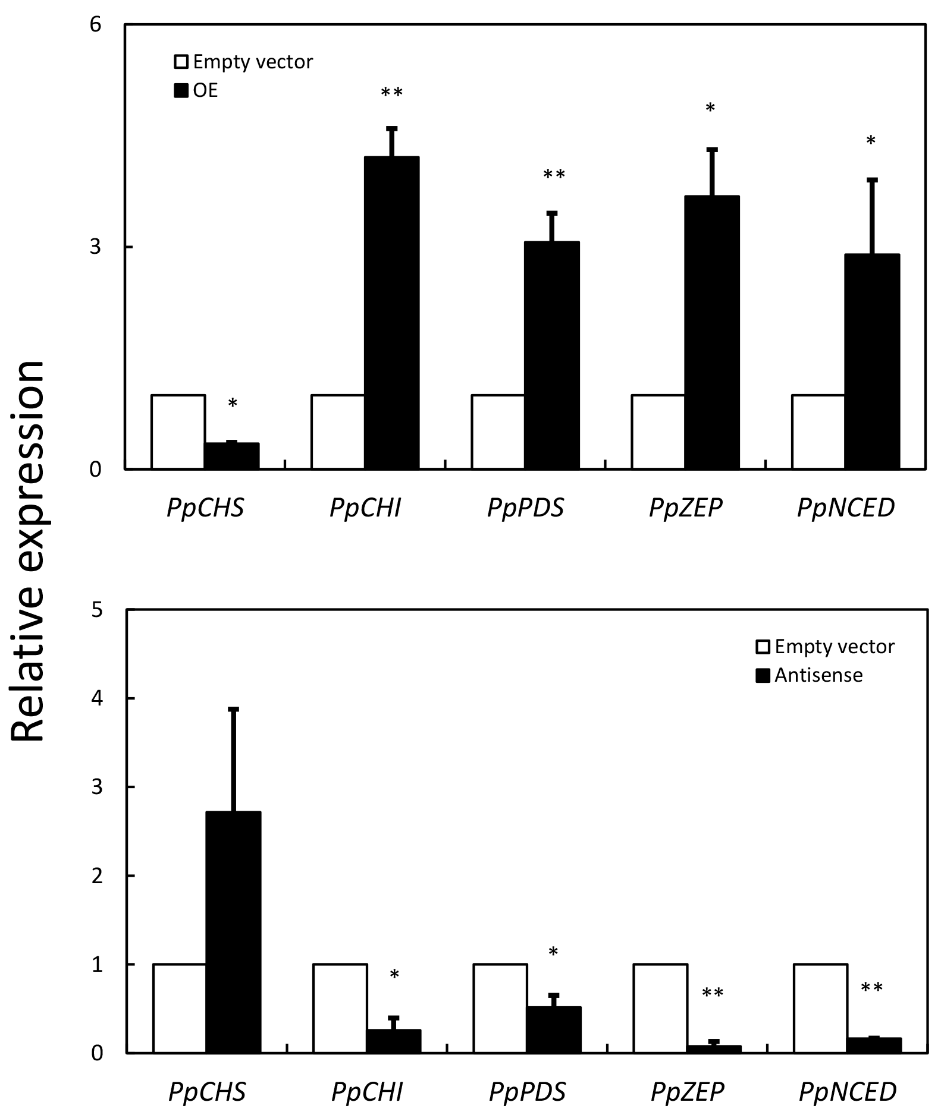


**Figure S6** PpERF.A16 positively regulates the expression of several ripening-related genes. The mRNA levels of *PpCHI*, *PpPDS*, *PpZEP*, and *PpNECD* genes were up-regulated in the peach fruits over-expressing *PpERF.A16* and down-regulated in the fruits silencing PpERF.A16 compared to the fruits infiltrated with an empty vector. *PpCHS* and *PpCHI* encode enzymes for flavonoid metabolism, *PpPDS* for carotenoid metabolism, and *PpZEP* and *PpNECD* for abscisic acid biosynthesis. Means and standard errors were calculated from six biological replicates. Single and double asterisks represent the levels of significance at *P* < 0.05 and <0.01, respectively.


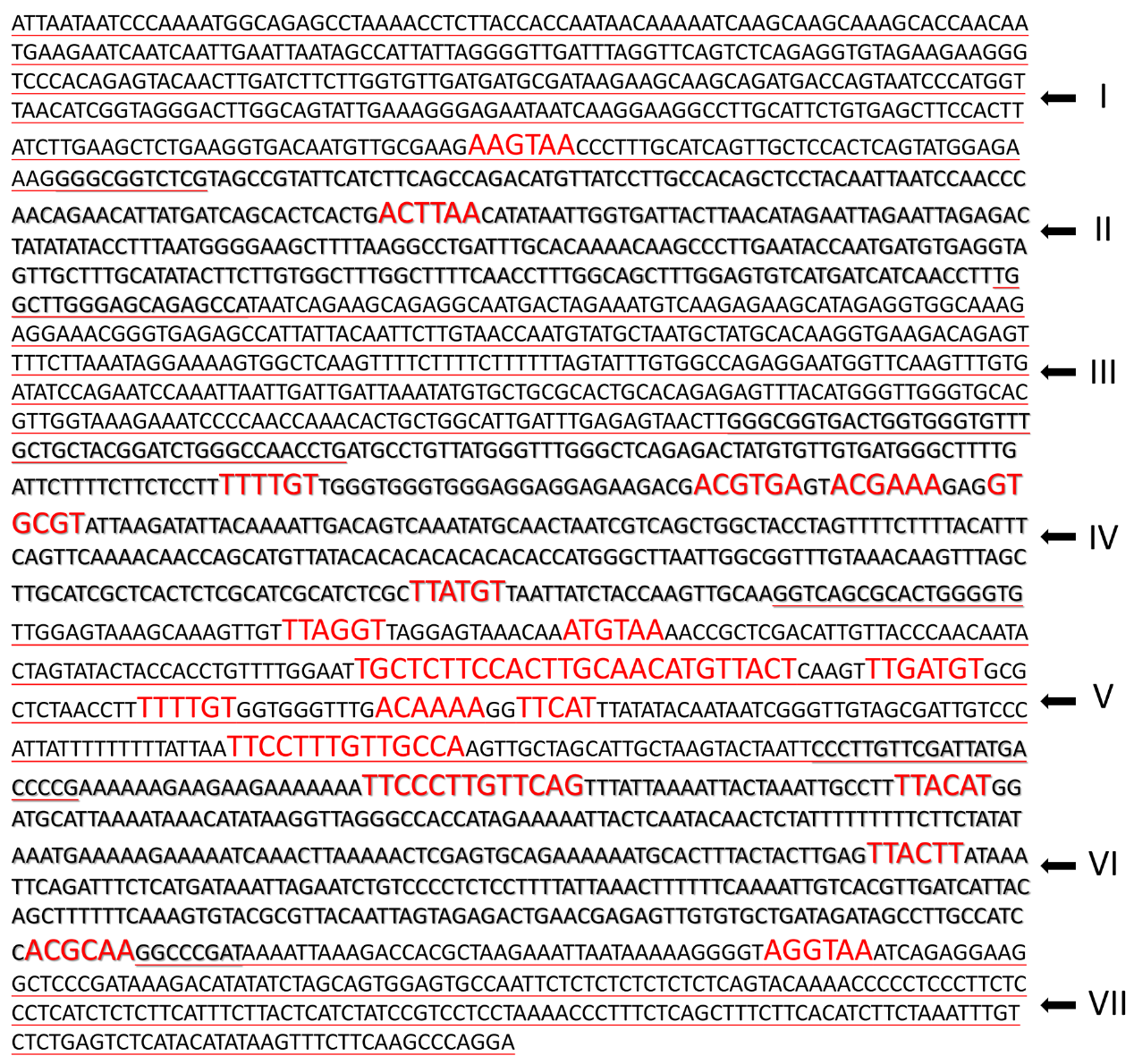


**Figure S7** *Cis*-regulatory elements for NAC TFs were predicted in the *PpERF.A16* promoter. The bases with red color are the NAC-binding elements.


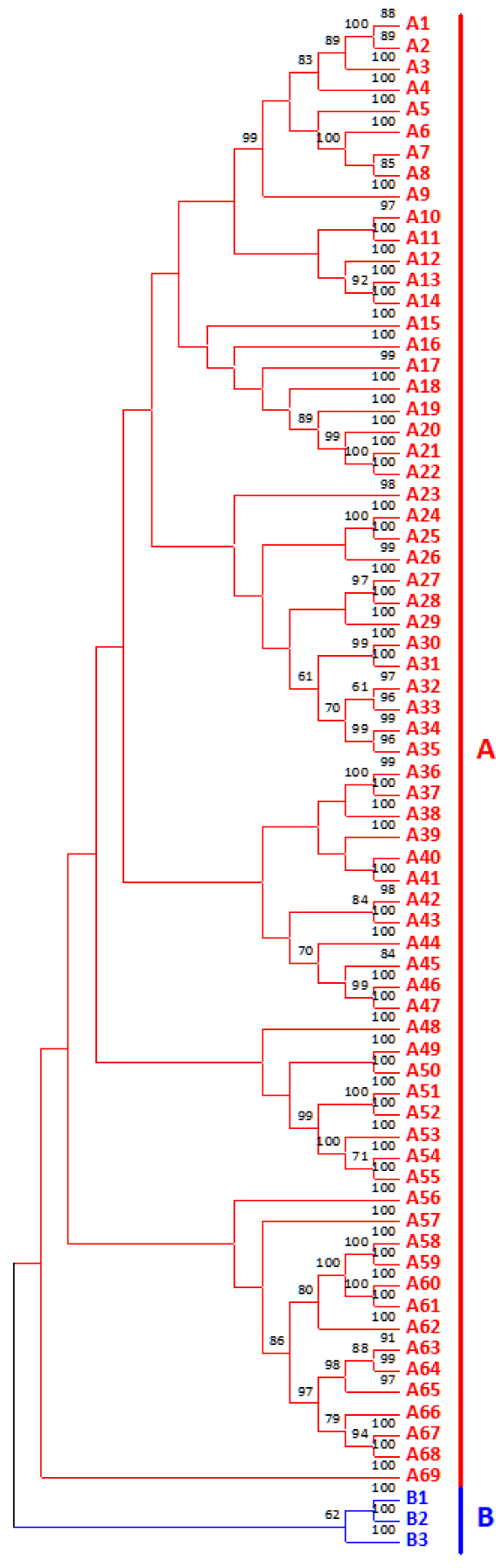


**Figure S8** Phylogenetic classification of *NAC* genes in peach and other fruit trees. Accession numbers of these genes are listed in Table S2.


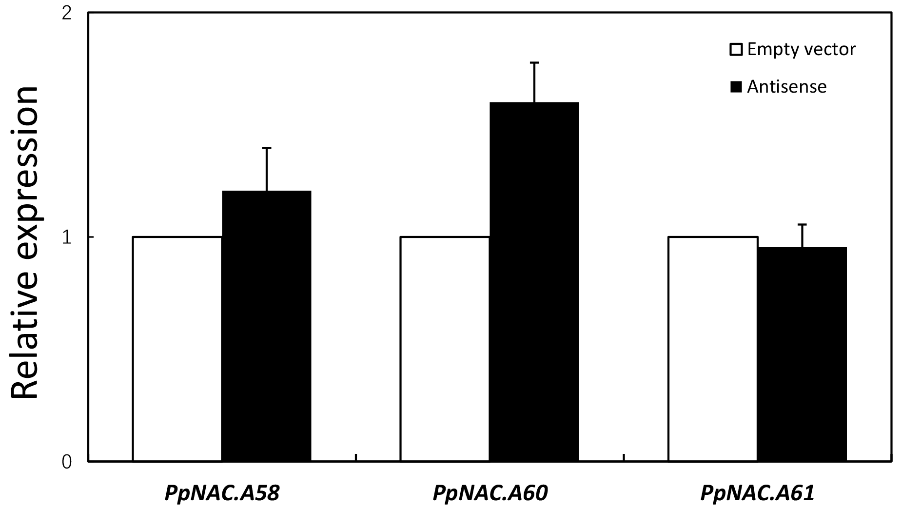


**Figure S9** Expression level of genes closely related to *PpNAC.A59* in the fruit silencing *PpNAC.A59*. Due to the extremely high bootstrap value (100) among A58, A59, A60, and A61 (Figure S8), the members of these four group in peach were tested in the fruit silencing *PpNAC.A59*. Thus, *PpNAC.A58*, *PpNAC.A60*, and *PpNAC.A61* were tested by qRT-PCR. The result showed that these three genes were not differentially expressed in the fruit silencing PpERF.A16 compared to the control.


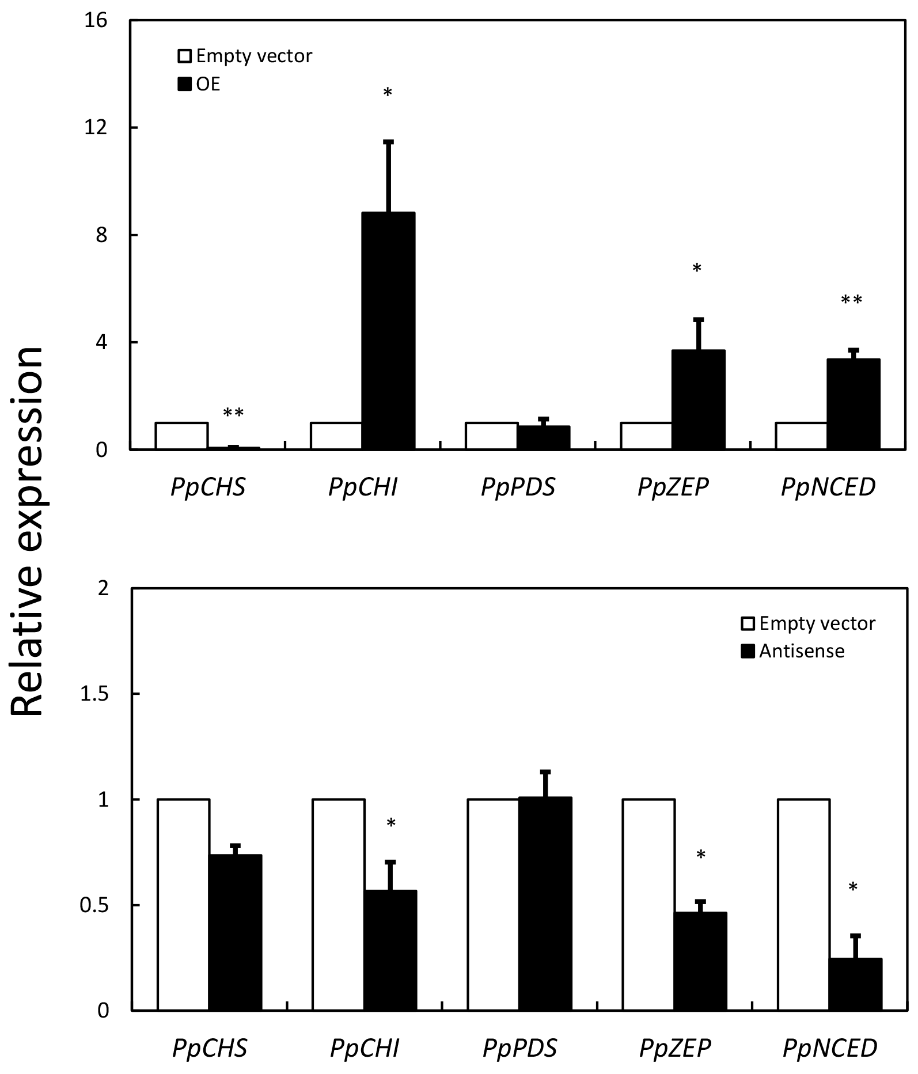


**Figure S10** PpNAC.A59 positively regulates the expression of several ripening-related genes. The mRNA levels of *PpCHI*, *PpZEP*, and *PpNECD* genes were up-regulated in the peach fruits over-expressing *PpNAC.A59* and down-regulated in the fruits silencing PpERF.A16 compared to the fruits infiltrated with an empty vector. *PpCHS* and *PpCHI* encode enzymes for flavonoid metabolism, *PpPDS* for carotenoid metabolism, and *PpZEP* and *PpNECD* for abscisic acid biosynthesis. Means and standard errors were calculated from six biological replicates. Single and double asterisks represent significance levels at *P* < 0.05 and <0.01, respectively.


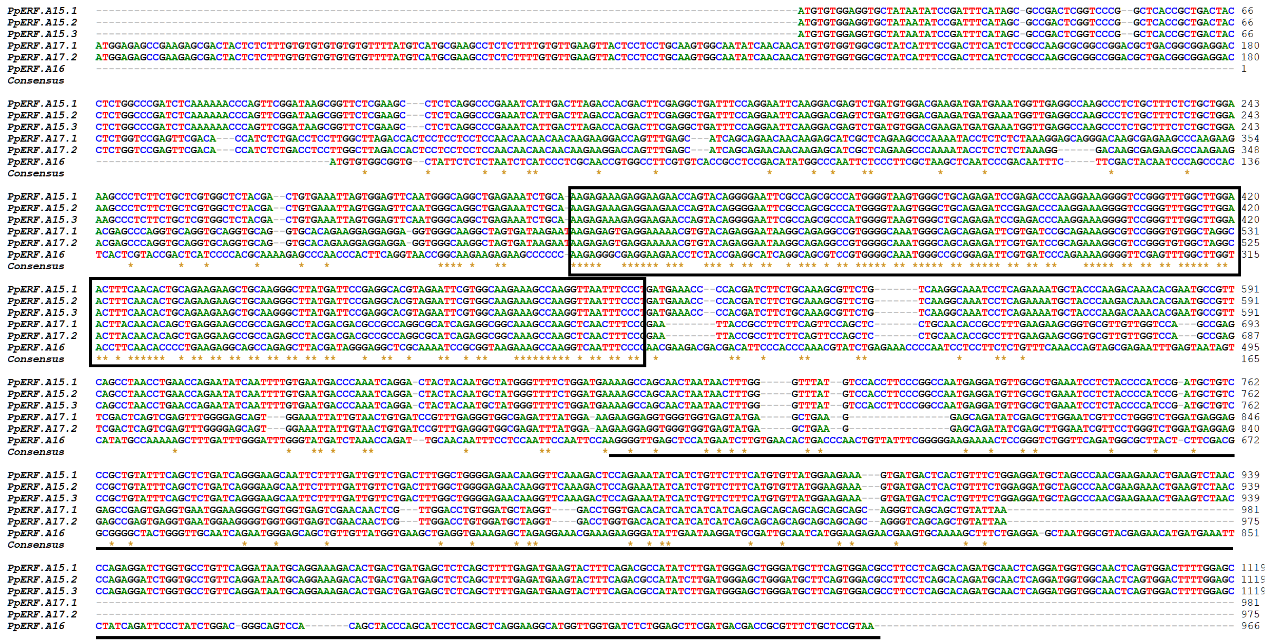


**Figure S11** Identification of the conserved and non-conserved regions among *PpERF.A16*, *PpERF.A15.1*, *PpERF.A15.2*, *PpERF.A15.3*, *PpERF.A17.1*, and *PpERF.A17.2*. The conserved region was boxed and the underlined sequences of *PpERF.A16* were inserted into pSAK277 vector to create a gene silencing construct.


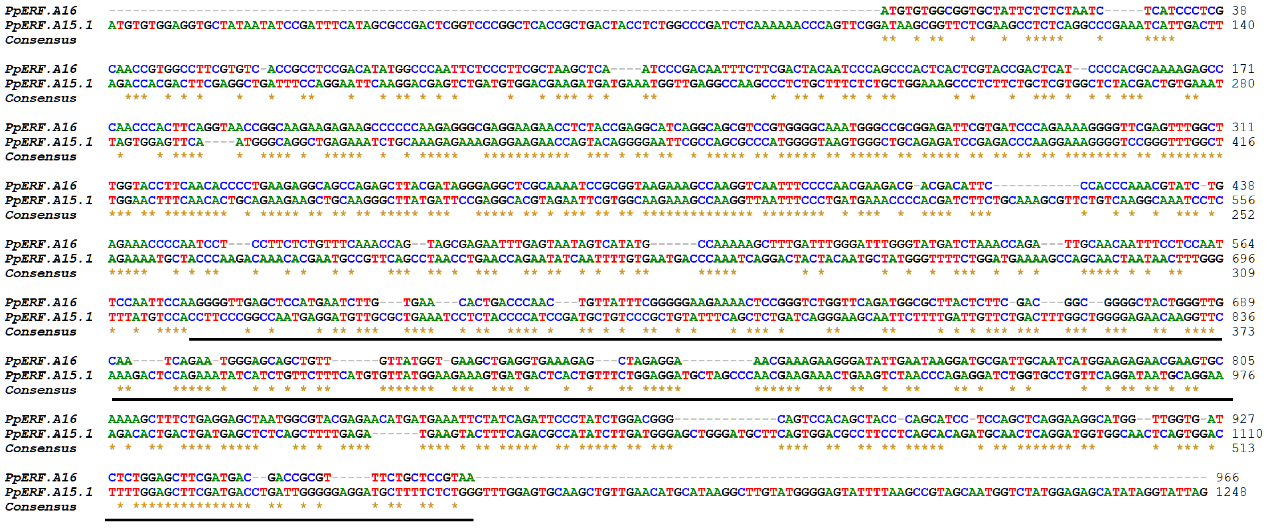


**Figure S12** Sequence alignment of *PpERF.A16* and *PpERF.A15.1*. The underlined sequences of *PpERF.A16* were inserted into pSAK277 vector to create a gene silencing construct.


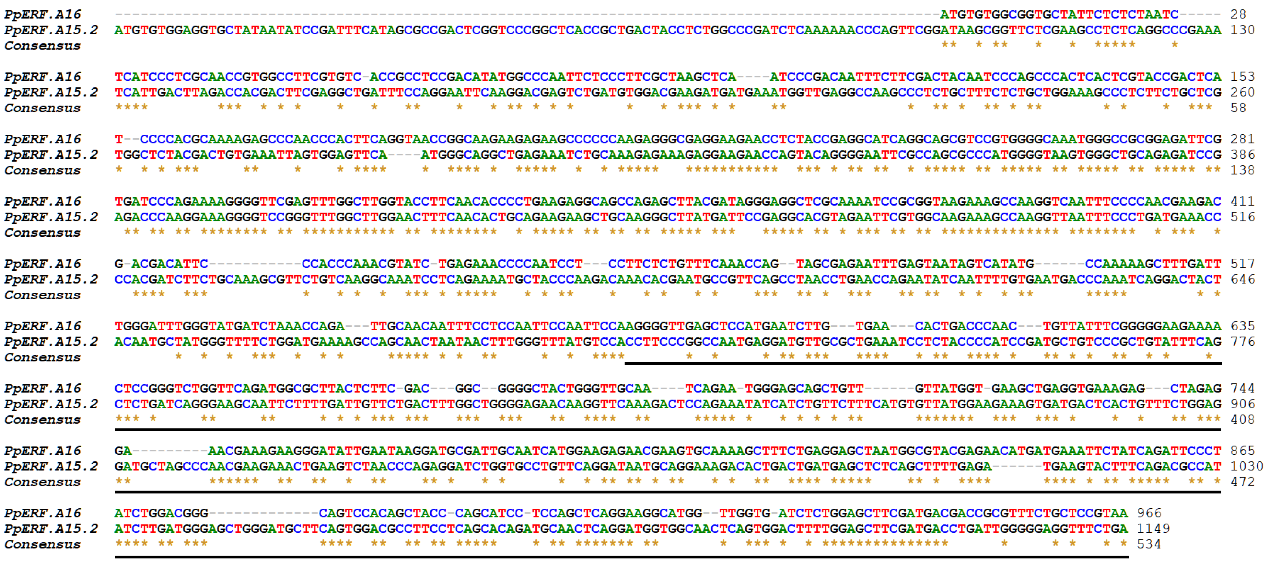


**Figure S13** Sequence alignment of *PpERF.A16* and *PpERF.A15.2*. The underlined sequences of *PpERF.A16* were inserted into pSAK277 vector to create a gene silencing construct.


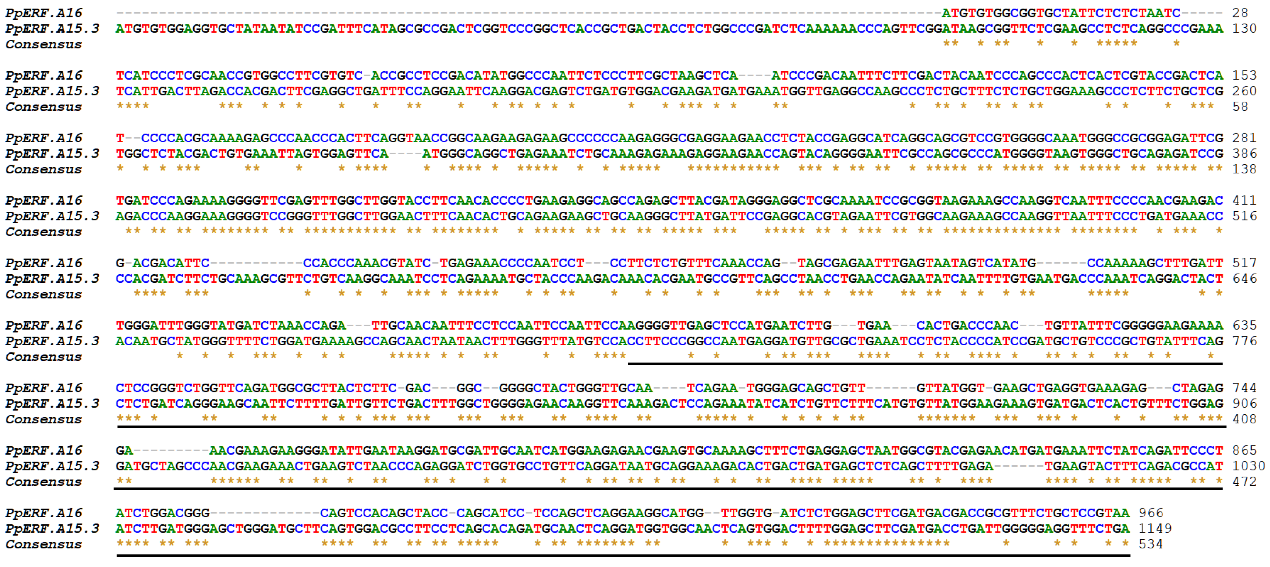


**Figure S14** Sequence alignment of *PpERF.A16* and *PpERF.A15.3*. The underlined sequences of *PpERF.A16* were inserted into pSAK277 vector to create a gene silencing construct.


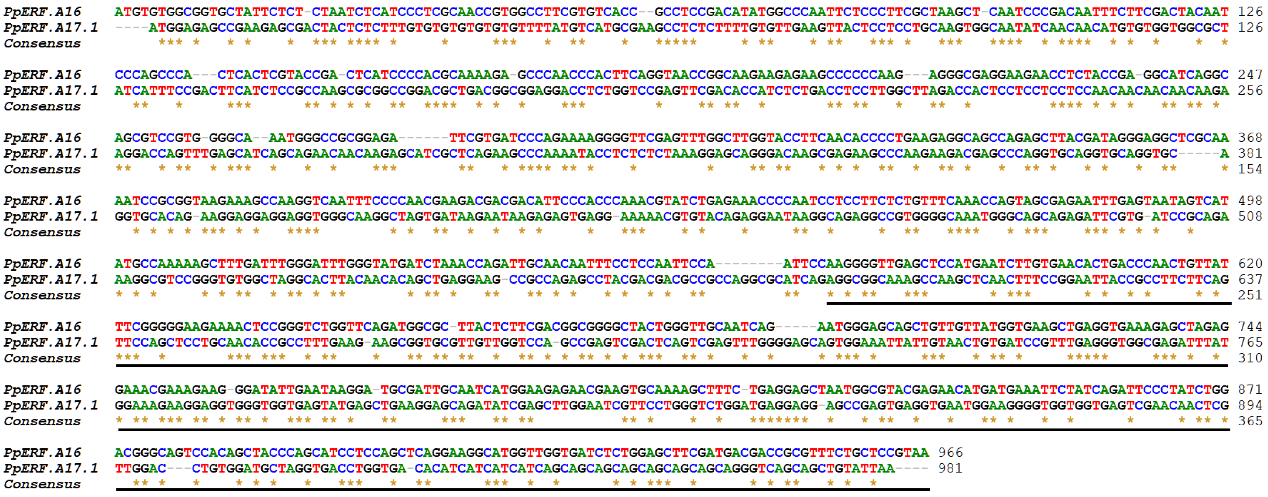


**Figure S15** Sequence alignment of *PpERF.A16* and *PpERF.A17.1*. The underlined sequences of *PpERF.A16* were inserted into pSAK277 vector to create a gene silencing construct.


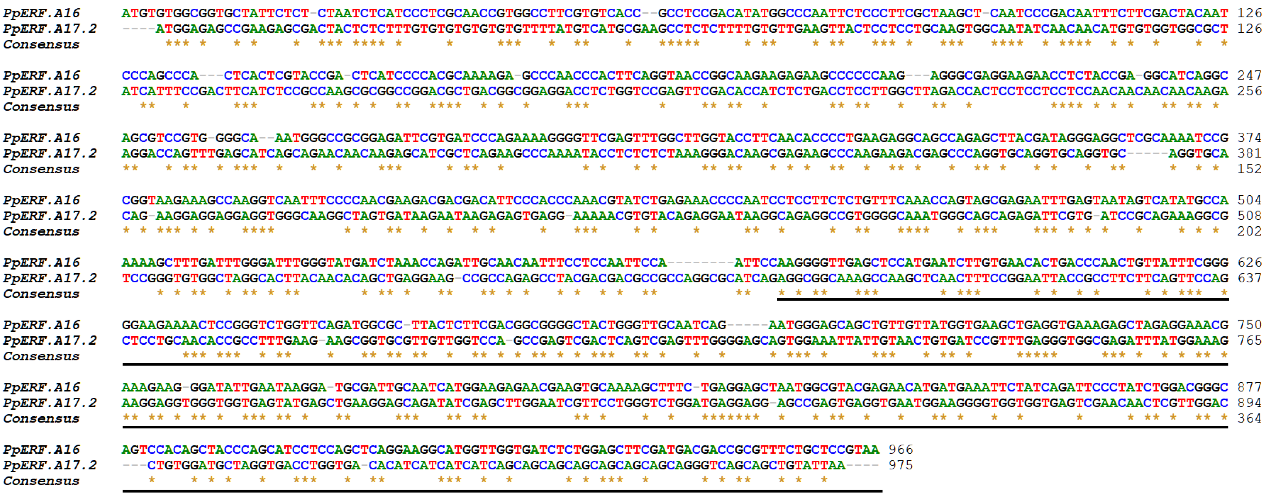


**Figure S16** Sequence alignment of *PpERF.A16* and *PpERF.A17.2*. The underlined sequences of *PpERF.A16* were inserted into pSAK277 vector to create a gene silencing construct.


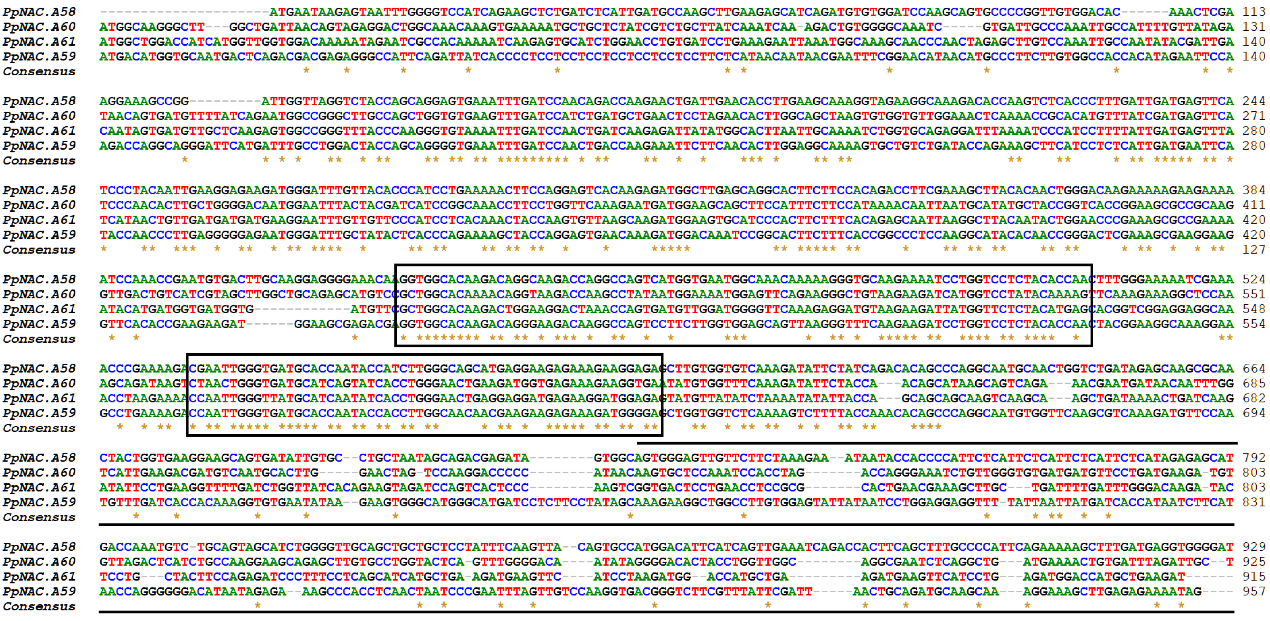


**Figure S17** Identification of the conserved and non-conserved regions among *PpNAC.A58*, *PpNAC.A59*, *PpNAC.A60*, and *PpNAC.A61*. The conserved region was boxed and the underlined sequences of *PpNAC.A59* were inserted into pSAK277 vector to create a gene silencing construct.


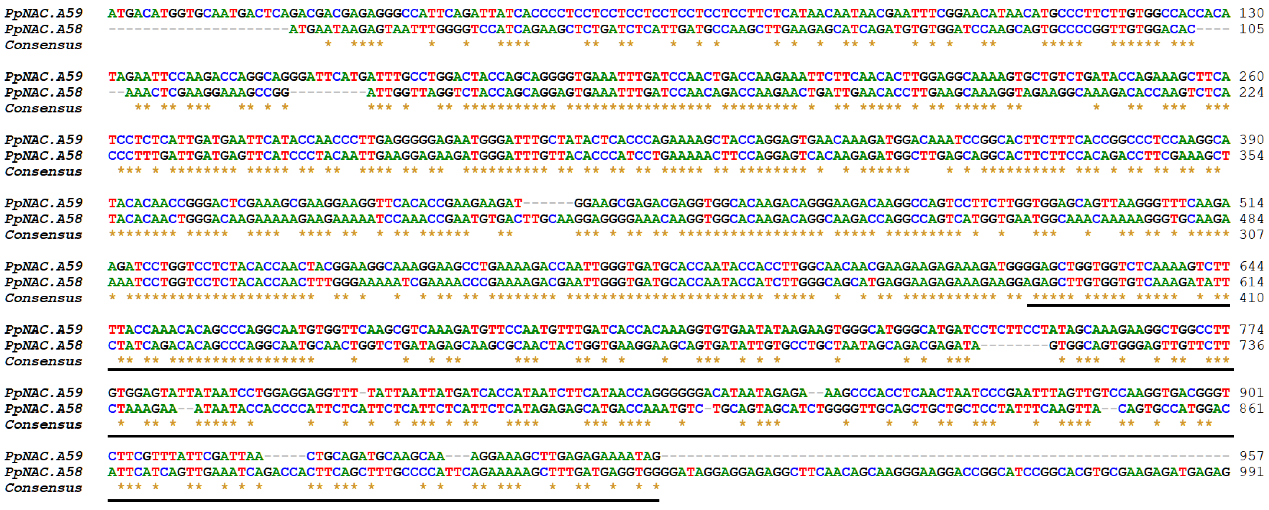


**Figure S18** Sequence alignment of *PpNAC.A59* and *PpNAC.A58*. The underlined sequences of *PpNAC.A59* were inserted into pSAK277 vector to create a gene silencing construct.


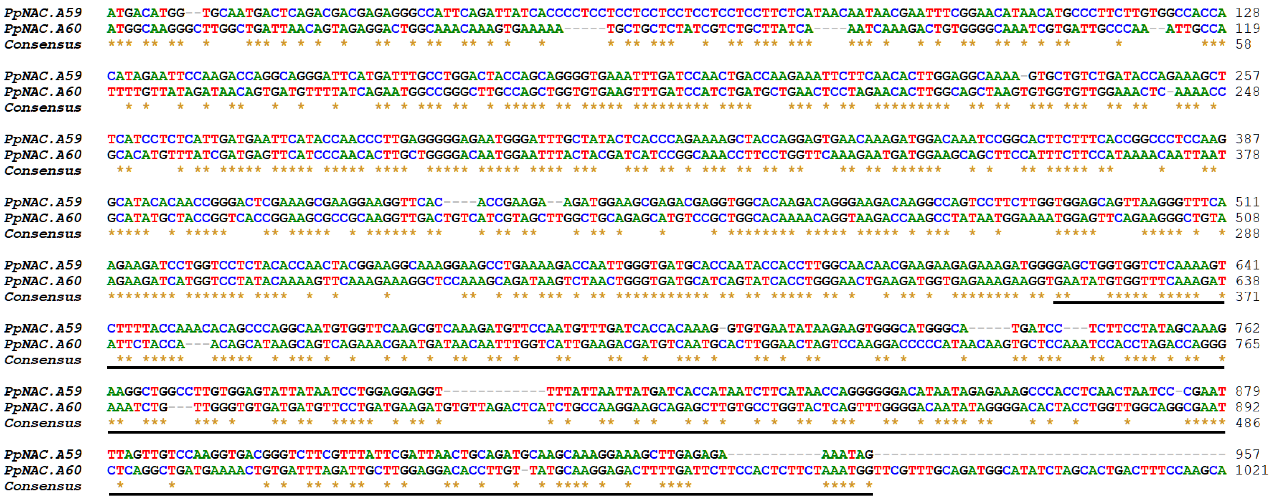


**Figure S19** Sequence alignment of *PpNAC.A59* and *PpNAC.A60*. The underlined sequences of *PpNAC.A59* were inserted into pSAK277 vector to create a gene silencing construct.


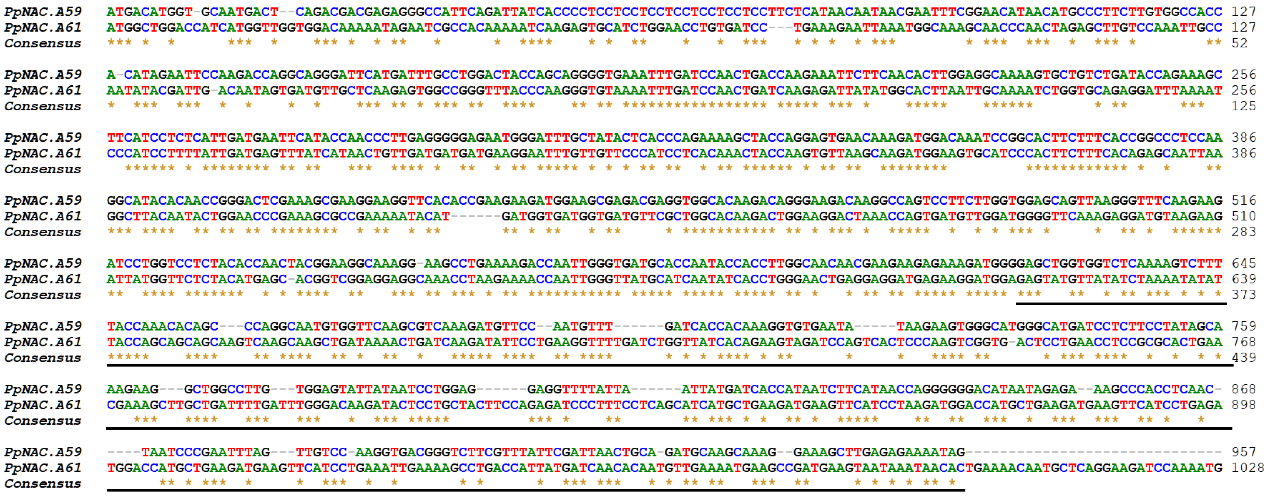


**Figure S20** Sequence alignment of *PpNAC.A59* and *PpNAC.A61*. The underlined sequences of *PpNAC.A59* were inserted into pSAK277 vector to create a gene silencing construct.
